# Characterization of Antibodies against Receptor Activity-Modifying Protein 1 (RAMP1): A Cautionary Tale

**DOI:** 10.3390/ijms232416035

**Published:** 2022-12-16

**Authors:** Erica R. Hendrikse, Tayla A. Rees, Zoe Tasma, Michael L. Garelja, Andrew Siow, Paul W. R. Harris, John B. Pawlak, Kathleen M. Caron, Elizabeth S. Blakeney, Andrew F. Russo, Levi P. Sowers, Thomas A. Lutz, Christelle Le Foll, Christopher S. Walker, Debbie L. Hay

**Affiliations:** 1 School of Biological Sciences, The University of Auckland, Auckland 1010, New Zealand; 2 Department of Pharmacology and Toxicology, The University of Otago, Dunedin 9016, New Zealand; 3 School of Chemical Sciences, The University of Auckland, Auckland 1010, New Zealand; 4 Maurice Wilkins Centre for Molecular Biodiscovery, The University of Auckland, Auckland 1010, New Zealand; 5 Department of Cell Biology and Physiology, University of North Carolina at Chapel Hill, Chapel Hill, NC 27599, USA; 6 Department of Molecular Physiology and Biophysics, University of Iowa, Iowa City, IA 52242, USA; 7 Center for the Prevention and Treatment of Visual Loss, Veterans Administration Health Center, Iowa City, IA 52246, USA; 8 Institute of Veterinary Physiology, University of Zurich, Zurich 8057, Switzerland

**Keywords:** RAMP1, brain, CGRP, migraine, amylin, GPCR, antibody validation

## Abstract

Calcitonin gene-related peptide (CGRP) is a key component of migraine pathophysiology, yielding effective migraine therapeutics. CGRP receptors contain a core accessory protein subunit: receptor activity-modifying protein 1 (RAMP1). Understanding of RAMP1 expression is incomplete, partly due to the challenges in identifying specific and validated antibody tools. We profiled antibodies for immunodetection of RAMP1 using Western blotting, immunocytochemistry and immunohistochemistry, including using RAMP1 knockout mouse tissue. Most antibodies could detect RAMP1 in Western blotting and immunocytochemistry using transfected cells. Two antibodies (844, ab256575) could detect a RAMP1-like band in Western blots of rodent brain but not RAMP1 knockout mice. However, cross-reactivity with other proteins was evident for all antibodies. This cross-reactivity prevented clear conclusions about RAMP1 anatomical localization, as each antibody detected a distinct pattern of immunoreactivity in rodent brain. We cannot confidently attribute immunoreactivity produced by RAMP1 antibodies (including 844) to the presence of RAMP1 protein in immunohistochemical applications in brain tissue. RAMP1 expression in brain and other tissues therefore needs to be revisited using RAMP1 antibodies that have been comprehensively validated using multiple strategies to establish multiple lines of convincing evidence. As RAMP1 is important for other GPCR/ligand pairings, our results have broader significance beyond the CGRP field.

## 1. Introduction

Calcitonin gene-related peptide (CGRP) is a neuropeptide with a role in headache and migraine. Multiple effective therapeutics targeting the CGRP system have been developed. However, these only treat a proportion of people living with migraine. Furthermore, side-effects, such as constipation, that have emerged with real-world therapy are not well understood. Understanding the complexity in the biological factors driving migraine pathophysiology and the mechanisms underlying the actions of CGRP would be beneficial [1].

Research into the actions of CGRP has focused on the CGRP receptor, comprising the calcitonin receptor-like receptor (CLR) and receptor activity-modifying protein 1 (RAMP1) [2]. However, RAMP1 can also heterodimerize with the calcitonin receptor (CTR) to form the AMY_1_ receptor, which is potently activated by both CGRP and a second member of the CGRP peptide family, amylin [2,3]. An amylin analogue, pramlintide, can induce migraine-like attacks, implicating the AMY_1_ receptor in migraine [4]. Therefore, the RAMP1 subunit is a critical and defining constituent of two migraine-relevant receptors. In addition, RAMP1 is reported to interact with a wide variety of other GPCRs (e.g., vasoactive intestinal polypeptide 2 receptor, glucagon receptors) to alter their pharmacological and physiological responses [5,6,7,8].

Despite its importance, our understanding of RAMP1 expression is limited. The presence of extensive high-affinity CGRP binding sites in discrete locations throughout the brain, including migraine-relevant regions such as the brainstem [9,10,11], implies that RAMP1 is present but does not directly demonstrate this. RNA studies report high levels of RAMP1 in a variety of brain regions, although its distribution varies between anatomical regions, species and studies [12,13,14,15]. However, the spatial distribution of mRNA does not always correspond to protein expression, particularly for neurons, where this may differ between cell bodies and projections [16,17].

Several studies have reported RAMP1 protein in nervous tissue, commonly detected using immunohistochemistry [3,18,19,20]. Examples of RAMP1 antibodies used in nervous tissue are provided in Table S1. It is evident in this table that much of this work has relied on two anti-RAMP1 antibodies (844 and 3158; see descriptions in Supplemental Information Table S2) that are not commercially available, or others that are no longer available for purchase, such as the work performed by Lennerz and colleagues [20]. This makes it difficult for others to conduct their own investigations. Other RAMP1 antibodies are available from commercial sources but they generally lack validation. There is a great need for comprehensively validated, widely available RAMP1 antibodies to allow unambiguous immunohistochemical mapping of RAMP1 protein expression, and analysis of its co-localization with GPCRs. This would substantially advance our understanding of the receptor-based mechanisms driving CGRP and amylin actions in migraine-relevant tissues and in other systems.

This study comprehensively profiled commercially available anti-RAMP1 antibodies alongside the 844 and 3158 antibodies to provide a comparison of the performance of different RAMP1 antibodies in detecting RAMP1 expression, focusing on rodent models. To enable direct comparisons to be made between them, antibodies (including 844 and 3158) were characterized in line with current antibody validation standards [21,22]. Multiple methods (immunocytochemistry [ICC], Western blotting and immunohistochemistry [IHC]) were employed, with the goal of unambiguously assigning antibody immunoreactivity to RAMP1, as opposed to off-target proteins. Antibodies were initially screened using a transfected cell system before a selection of antibodies were further profiled in rat and mouse tissue, followed by tissue from mouse models with genetic manipulation of RAMP1 expression, including RAMP1 knockout (KO) mice.

## 2. Results

### 2.1. Anti-RAMP1 Antibodies Detected a Range of Bands in Transfected Cell Western Blots

**Figure 1:**
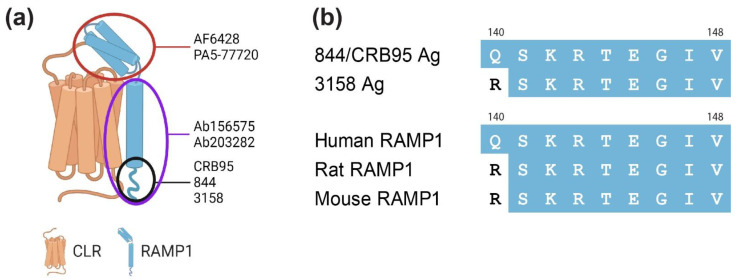
Location of antigenic sequences within RAMP1 for the antibodies used. (**a**) Colored ovals indicate the location of the antigenic sequence: red, within the extracellular domain, purple, within the transmembrane and C-terminal domains, and black, within the C-terminus alone. (**b**) Alignment of the known antigenic sequences within the C-terminal domains compared to human, rat and mouse C-termini (residues 140–148). Residues that are identical to the human RAMP1 C-terminus are shown in blue with white text, residues which are not identical are shown in white with black text. Alignment was performed in Clustal Omega 1. Numbering refers to the human RAMP1 sequence. Ag = Antigenic sequence, CLR = calcitonin receptor-like receptor, RAMP1 = receptor activity-modifying protein.

Antibodies against RAMP1 were selected from those that are commonly used, depending on their availability (Table S1). Antibodies targeting RAMP1 were initially screened by Western blotting. When conducting these experiments, we had the following assumptions. Firstly, we expected that antibodies able to detect RAMP1 in Western blots would produce a band consistent with the expected molecular weight of RAMP1. Secondly, given the high level of overall amino acid sequence identity between human, rat and mouse RAMP1 (~69%, Figure S1), we expected that RAMP1 antibodies would be likely to detect each of these species, especially antibodies that are raised against the C-terminal region (844, 3158; Figure 1).

We first confirmed the molecular characteristics of human RAMP1 by conducting Western blotting with two different N-terminally epitope-tagged forms of the protein (Figure S2a). This allowed us to use highly specific anti-tag antibodies and gave us reference molecular weights for subsequent experiments. Consistent with previous studies, two bands at apparent molecular weights of ~14 kDa and ~28 kDa were observed (Figure S2b), which likely correspond to RAMP1 monomer and dimers, respectively [23,24]. In the figures, we have marked the bands that correspond to the monomer with black arrowheads, and the bands that likely correspond to the dimer with red arrowheads. Due to issues with product availability, we compared multiple protein ladders and saw that band sizes in the Benchmark ladder were inaccurate compared to the Abcam ladder (Figure S2c, [25]). Consequently, the apparent molecular weights referred to in the text are estimates based on the Abcam protein ladder.

**Figure 2:**
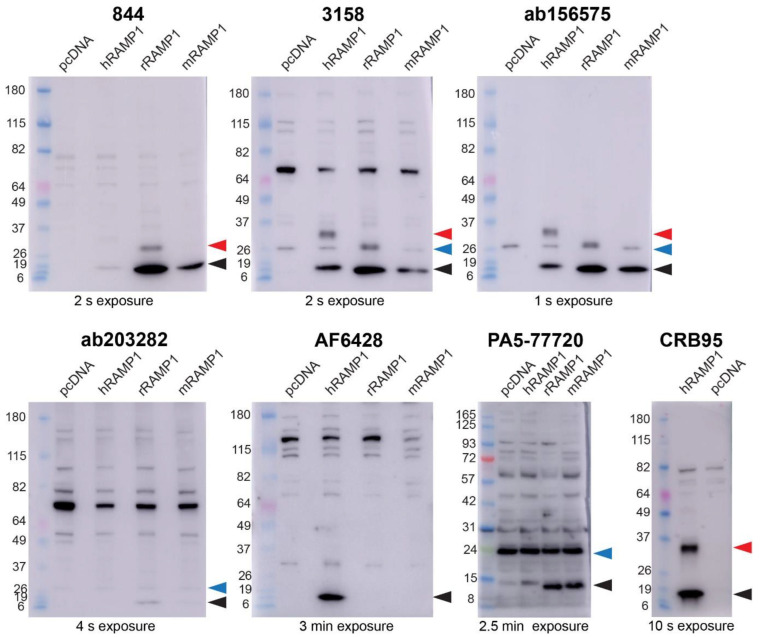
Immunoblotting of transfected cell preparations with anti-RAMP1 antibodies. Membrane-enriched protein samples were prepared from HEK293S cells transfected with hCLR:myc-hRAMP1, rCLR:rRAMP1, mCLR:mRAMP1 or vector. Blots were probed with 844 (5 µg/mL), 3158 (1:200), ab156575 (0.612 µg/mL), ab203282 (1 µg/mL), AF6428 (1 µg/mL), PA5-77720 (0.85 µg/mL) or CRB95 (2 µg/mL) primary antibody. Exposure time is indicated under each blot. Blots are representative of three independent experiments using the Benchmark protein ladder with MOPS buffer, except PA5-77720 which used MES buffer and the Abcam protein ladder. Note that, using these methods, the Benchmark protein ladder over-estimates molecular weights (see Figure S2). Black-filled arrowheads indicate the predicted monomeric RAMP1 band; red-filled arrowheads indicate likely RAMP1 dimers; blue-filled arrowheads mark the non-specific ~24 kDa band described in the text.

The antibodies against RAMP1 were then tested using membrane-enriched protein preparations containing human myc-RAMP1, or untagged rat and mouse RAMP1, and compared to vector control lysates. The use of tagged human RAMP1 enabled direct comparison to experiments that used the myc antibody, as described above. All antibodies produced multiple bands that varied in their molecular weight and intensity (Figure 2). The 844, 3158 and ab156575 antibodies produced bands at a molecular weight consistent with a human, rat and mouse RAMP1 monomer (~14 kDa), in the corresponding samples. For 844, samples containing human RAMP1 produced a fainter band compared with rodent RAMP1. For ab156575, a ~28 kDa band consistent with a human RAMP1 dimer was present. This was also observed in two of the three blots probed with 3158. For 844, a band representing a probable rat RAMP1 dimer was observed at ~24 kDa. This difference in dimer size for human and rat is likely due to the presence of the myc-tag on hRAMP1, with untagged human and rat RAMP1 displaying similar dimeric band sizes of ~25 kDa (Figure S3).

For the 3158 and ab156575 antibodies, an additional, likely non-specific, band was also observed at ~24 kDa across all lanes, including the vector control (ladder shows ~26 kDa; note that, using these methods, the Benchmark protein ladder over-estimates the molecular weights as shown in Figure S2). We have marked this non-specific band with a blue arrowhead. Given the proximity of this band to the potential rat RAMP1 dimer band, and the potential difficulty in distinguishing these bands depending on the conditions used, additional experiments were conducted with 844 and ab156575 using MES buffer to obtain enhanced band separation (Figure S3). For ab156575, the band at ~24 kDa (blue) can be clearly distinguished from the likely rat RAMP1 dimer band (red), confirming its non-specific nature. This illustrates the importance of the choice of ladder, membrane, gel and buffer as this non-specific band could easily be mistaken for a RAMP1 dimer in tissue samples.

Anti-RAMP1 ab203282 and PA5-77720 detected bands consistent with the molecular weights of rat and mouse RAMP1 monomers but did not appear to detect human RAMP1 (Figure 2 and Figure S4). In contrast, AF6428 only produced a RAMP1-like band in samples containing human RAMP1 protein. These antibodies generated a range of likely non-specific bands at multiple sizes across all lanes, particularly the PA5-77720 antibody, which had an intense non-specific band at ~24 kDa. The PA5-77720 antibody also produced high variability between independent experiments, with variation in the number and size of the bands observed. CRB95, which was raised using the same antigenic sequence as 844, was only examined for the detection of human RAMP1. Bands were observed, consistent with a predicted RAMP1 monomer and dimer. Western blot results under the conditions tested are summarized in Table 1. Additional optimization of experimental conditions could be considered in future experiments to focus on detecting specific forms of RAMP1, such as dimer bands or RAMP1 from specific species.

### 2.2. Anti-RAMP1 Antibodies Produced Immunoreactivity in RAMP1-Transfected Cells Using ICC

Using multiple methods to screen antibodies increases confidence in antibody specificity [21,26]. Therefore, ICC was employed to further evaluate the anti-RAMP1 antibodies. ICC also provides information about the compatibility of antibodies with fixed samples and therefore is a useful screening tool when IHC studies are planned. We first confirmed the suitability of our methods by using an anti-myc antibody to detect the myc tag on human RAMP1. This resulted in strong detection of myc-tagged human RAMP1 (Figure S5).

**Figure 3:**
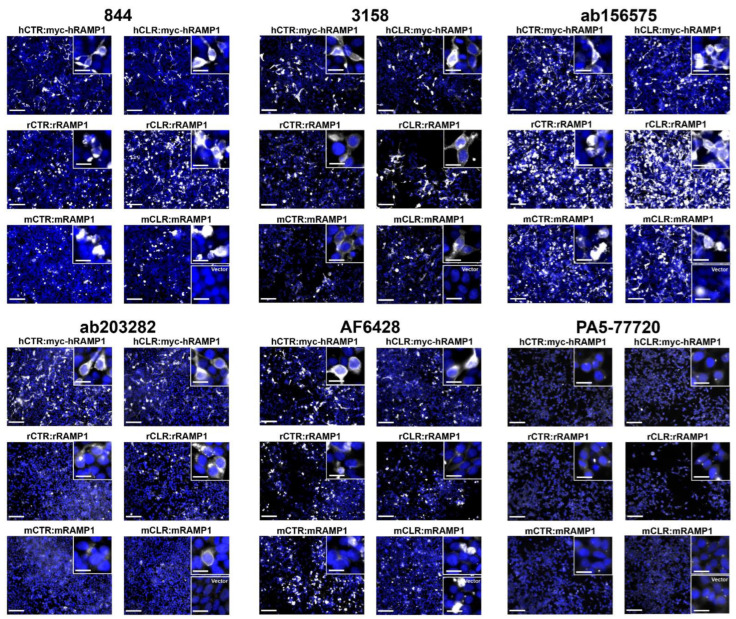
Immunofluorescent ICC of transfected HEK293S cells using anti-RAMP1 antibodies. Immunoreactivity of 844 (5 µg/mL), 3158 (1:500), ab156575 (3.06 µg/mL), ab203282 (5 µg/mL), AF6428 (1 µg/mL), and PA5-77720 (4.25 µg/mL) is shown in greyscale and nuclear DAPI staining in blue. Insets display immunoreactivity for each transfection condition with greater magnification. For each antibody, the lower inset in mCLR:mRAMP1 shows an example of immunoreactivity in the vector control. Images are representative of two (PA5-77720) or three (844, 3158, ab156575, ab203282, AF6428) independent experiments. Scale bars represent 100 µm or 20 µm for insets.

For testing the native RAMP1 antibodies, we used human, rat and mouse receptors comprising CLR or CTR with RAMP1. Anti-RAMP1 antibodies 844, 3158, ab156575, ab203282, and AF6428 produced immunoreactivity in RAMP1-transfected cells, compared to the signal observed for vector-transfected controls (Figure 3). There was some variability in the detection of RAMP1 of different species observed, depending on the antibody. For example, 844, 3158 and ab156575 exhibited immunoreactivity in cells transfected with human, rat, and mouse RAMP1. In contrast, ab203282 displayed robust immunoreactivity with human RAMP1, weaker immunoreactivity with rat RAMP1, and very weak, infrequent immunoreactivity with mouse RAMP1 (Figure 3). AF6428 only appeared to generate immunoreactivity in cells transfected with human RAMP1. However, this antibody produced inconsistent results between replicates and displayed intense non-specific clumps, which made it difficult to define positively stained cells (Figure 3 and Figure S6). PA5-77720 did not appear to detect human, rat or mouse RAMP1, under the conditions we used, with no immunoreactivity observed above signal from the vector-transfected control (Figure 3). Additionally, no specific RAMP1-like immunoreactivity was observed with a higher PA5-77720 concentration (Figure S7). All ICC experiments were performed without antigen retrieval. This could be considered in future experiments, particularly for PA5-77720. CRB95 appeared to detect human and rat RAMP1; we did not test this antibody against mouse RAMP1 (Figure S8). Although results from this antibody seemed promising, even under the storage conditions recommended by the manufacturer, CRB95 produced inconsistent results which may be related to its stability. An additional observation was that cells transfected with rat or mouse RAMP1 displayed a more rounded morphology than those transfected with human RAMP1, which was visible through the immunoreactivity (Figure 3).

Cross-reactivity with human RAMP2 or RAMP3 was also examined for antibodies 844, 3158, ab156575, AF6428 and CRB95 (Figure S6). No immunoreactivity above vector was observed in cells transfected with human RAMP2 or RAMP3 for any of the antibodies tested. However, non-specific clumps were present with AF6428, similar to that seen in the vector control cells.

### 2.3. Anti-RAMP1 Antibodies Produced Immunoreactivity in Rat Brain Using IHC

We chose to progress three antibodies into the next step of validation, IHC and Western blotting using rat tissue, which tests whether antibodies can detect endogenous levels of RAMP1 and allows comparison with previous RAMP1 studies. We chose 844, ab156575 and 3158 for these studies. This was based on their ability to detect human, rat and mouse RAMP1 in Western blots and ICC using transfected cells, their detection of fewer non-specific bands compared to other antibodies, and the widespread use of 844 in the literature. We did not use ab203282, AF6428 or PA5-77720 because they showed species specificity, which limits their translational value and/or they were not compatible with robust detection of RAMP1 across Western blotting and ICC.

**Figure 4:**
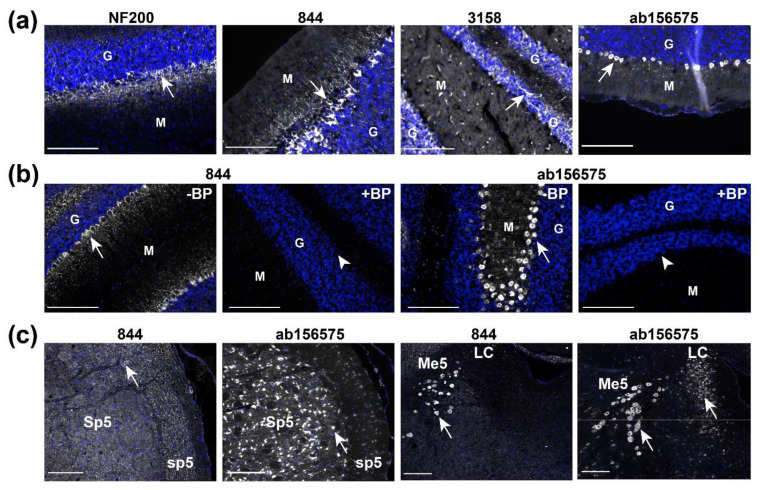
Neurofilament and RAMP1-like immunoreactivity in rat brain structures. (**a**) Immunoreactivity of NF200 (2.72 µg/mL), 844 (10 µg/mL), 3158 (1:200) and ab156575 (3.06 µg/mL) in rat cerebellum. (**b**) Immunoreactivity of 844 (10 µg/mL) and ab156575 (3.06 µg/mL) in the presence or absence of 50 µM blocking peptide (BP). (**c**) Immunoreactivity of anti-RAMP1 antibodies 844 (10 µg/mL) and ab156575 (3.06 µg/mL) in rat spinal trigeminal nucleus (Sp5), spinal tract of the trigeminal nerve (sp5), and locus coeruleus (LC). Anti-RAMP1 immunoreactivity is shown in greyscale and nuclear staining in blue. White arrows indicate positive immunoreactivity. White arrowheads indicate regions where immunoreactivity was absent. Scale bars represent 200 µm. (**a**) Images are representative of immunoreactivity detected in three individual rats. (**b**) Images are representative of immunoreactivity detected in three individual rats. (**c**) Sp5 images are representative of immunoreactivity detected in three (ab156575) or five (844) individual rats. LC images are representative of immunoreactivity detected in three (ab156575) or four (844) individual rats. Heat-induced epitope retrieval (HIER) was performed for ab156575. G = granular layer, M = molecular layer.

Prior data for RAMP1 expression in the brain have focused on the rat cerebellum, with both mRNA and protein studies suggesting the presence of RAMP1 [13,14,15,27]. The cerebellum also displays high-density binding of radiolabelled CGRP [9,10,28]. Therefore, the rat cerebellum was selected as a positive control tissue where we would expect to be able to detect RAMP1 using IHC. Anti-RAMP1 antibodies were accompanied by an anti-neurofilament (NF200) antibody, a neuronal marker, which confirmed that the tissue was of suitable quality (Figure 4a).

The three RAMP1 antibodies displayed distinct immunoreactivity in the rat cerebellum (Figure 4a). The antibody 844 produced a striking pattern of immunoreactivity in large neurons and their processes between the granular and molecular layers, which was consistent with previous studies [19,29]. In contrast, 3158 produced a capillary-like immunoreactive pattern throughout the tissue and immunoreactivity in the granular layer. This capillary-like pattern has previously been observed in the Sp5 of rat brain [30]. ab156575 produced immunoreactivity between the granular and molecular layers, like 844. However, the pattern of immunoreactivity was distinct from that of 844, with immunoreactivity from ab156575 largely restricted to rounded cell bodies rather than irregular cell bodies and processes like 844. Additionally, cell-like immunoreactivity was occasionally present in the granular layer and weak-diffuse staining in the molecular layer.

A ‘blocking peptide’ corresponding to the C-terminal RAMP1 sequence (844 antigen; Figure 1) was used to test whether the immunoreactivity for 844 and ab156575 was driven by an antibody-antigen interaction. As shown in Figure 4b and Figure S9, immunoreactivity generated by 844 in the cerebellum (and in transfected cells) was abolished when antibodies were pre-incubated with blocking peptide. The immunoreactivity for ab156575 was also abolished by the same blocking peptide. These data show that both antibodies bind to a target with a similar epitope to that present in the blocking peptide. However, these results do not confirm that the antibody is binding to RAMP1 specifically.

Additional migraine-relevant regions of the rat brain, the spinal trigeminal nucleus (Sp5) and tract (sp5), and locus coeruleus (LC), were examined for RAMP1-like immunoreactivity with 844 and ab156575 (Figure 4c) [20,31]. As in the cerebellum, the patterns of immunoreactivity observed in these regions also showed variation between these two antibodies. In Sp5/sp5, a speckled pattern consistent with fiber-like structures cut in transverse was produced by 844, whereas ab156575 appeared to produce immunoreactivity in cell bodies in Sp5 with little immunoreactivity in the tract (sp5). In the region of the LC, both antibodies produced immunoreactive signal in large cell bodies in the mesencephalic trigeminal nucleus (Me5). ab156575 also stained cell bodies in the LC; this immunoreactivity was not present with 844. Additional regions (area postrema (AP), nucleus of the solitary tract (NTS), and reticular nucleus of the medulla oblongata (Ret)) also showed distinct patterns of immunoreactivity between the two antibodies (Figure S10). Generally, 844 produced more fiber-like patterns, while ab156575 generated abundant immunoreactivity in cell bodies.

### 2.4. Anti-RAMP1 Antibodies Detected a Range of Bands in Rat Brain by Western Blotting

**Figure 5:**
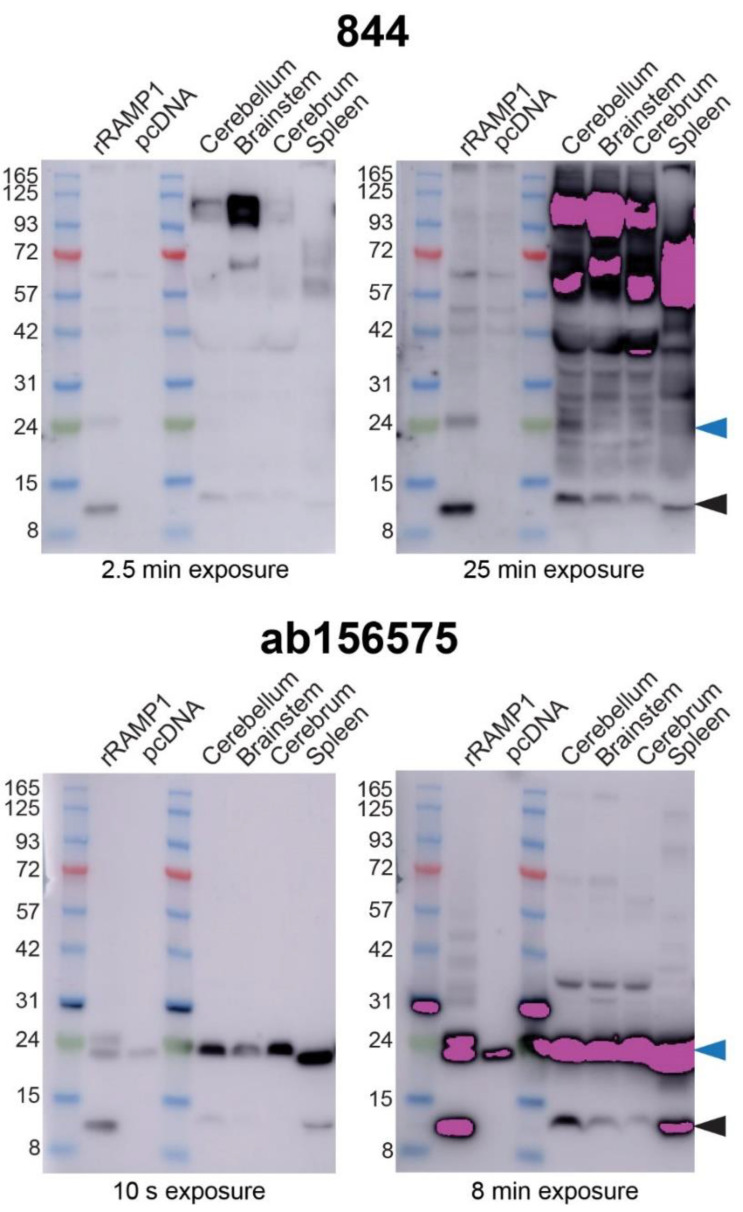
Immunoblotting of rat brain and spleen lysates with anti-RAMP1 antibodies. Lysates were prepared from HEK293S cells transfected with rCLR:rRAMP1 or vector, or rat tissue. Blots were probed with 844 (2 µg/mL) or ab156575 (0.612 µg/mL) primary antibody. Exposure times are indicated below each blot. Blots are representative of three independent experiments using MES running buffer and the Abcam protein ladder. Pink bands indicate signal above the range of the blot imager. Cross-reactivity is evident between a ladder protein and ab156575 in some blots. Black-filled arrowheads indicate the predicted monomeric RAMP1 band; blue-filled arrowheads mark the non-specific ~24 kDa band described in the text.

IHC showed that two anti-RAMP1 antibodies, that detected RAMP1 in transfected cells, yielded distinct patterns of immunoreactivity in multiple rat brain regions. We therefore compared 844 and ab156575 using Western blotting of tissue samples because this technique can provide molecular weight information and help delineate whether antibodies are genuinely detecting RAMP1 in rat brain. We generated Western blots using preparations of rat brain, divided into different brain regions (cerebellum, brainstem, cerebrum). Rat spleen was also used as this has previously shown high levels of RAMP1 expression [32]. Transfected cell preparations were used alongside the tissue samples to provide a positive control and allow comparison of bands across samples. Figure 5 shows the results and includes two exposure times because it was challenging to visualize high and low-intensity bands using a single exposure time.

Both 844 and ab156575 produced bands at ~14 kDa in lanes containing samples prepared from rat spleen or cells transfected with rRAMP1, consistent with the molecular weight of monomeric RAMP1 (Figure 5 and Figure S11) and similar to previous Western blots (Figure 2). For the transfected cells, the likely rRAMP1 dimer was present at ~24 kDa, along with the non-specific band for ab156575 at a similar apparent molecular weight, similar to previous blots (Figure 2). In samples prepared from rat brain, both antibodies detected a band at approximately the molecular weight of monomeric RAMP1, though the position of this corresponded with a slightly higher apparent molecular weight. This could mean that in brain tissue, a variant of RAMP1 is being detected, it has one or more post-translational modifications, or this is not actually RAMP1. For 844, a band consistent with a RAMP1 dimer could not be clearly distinguished in the tissue preparations because there were many bands detected. These additional bands were particularly intense at ~57 kDa and above, but ranged across the blots and were clearly visible with the extended exposure time. ab156575 produced fewer additional bands but the non-specific band at ~24 kDa confounded the ability to determine whether a RAMP1 dimer was present in rat brain and spleen preparations. Prolonged exposure revealed that blots probed with ab156575 cross-reacted with proteins in the Abcam ladder (Figure 5 and Figure S11).

We attempted to identify the protein present in the ~24 kDa band using mass spectrometry (methods described in Supplemental Information). As controls, samples were prepared from rat RAMP1 transfected HEK293S cells, rat spleen and rat cerebellum from between the 8 to 15 kDa markers. Rat RAMP1 was detected in the transfected HEK293S cell and rat spleen samples, indicating the presence of a RAMP1 monomer. However, no rat RAMP1 was detected in the rat cerebellum sample. RAMP1 expression may be below the limit of detection in this sample. No RAMP1 was detected in samples taken at the ~24 kDa marker in transfected HEK293S cells, rat cerebellum or brainstem, suggesting the absence of a RAMP1 dimer at this molecular weight. However, this is not conclusive as detecting proteins with low-level endogenous expression using mass spectrometry can be challenging, as seen with the absence of detection of the RAMP1 monomer in the cerebellum [33]. Although RAMP1 was not detected at this molecular weight, several other proteins were found in both the HEK293S and rat brain samples analyzed using mass spectrometry, which could be candidates for the cross-reactive protein(s) that underlies the non-specific ~24 kDa band (Table S3).

### 2.5. RAMP1 Antibodies Produced Different Immunofluorescence Patterns in Mouse Brain

The literature has predominantly investigated RAMP1 expression in rats but mice are also commonly used to investigate CGRP biology. Furthermore, several mouse models exist that have manipulated RAMP1 expression [34,35,36,37]. These offered an opportunity to further interrogate the specificity of the RAMP1 antibodies. We first characterized patterns of immunoreactivity for three antibodies (844, ab156575, 3158) in wildtype (WT) C57BL/6J mice (NZ colony), using three brain regions reported to highly express RAMP1, cerebellum, cerebrum, and nucleus accumbens (ACB) [13,15,38,39,40,41]. As with rat brain, NF200 immunoreactivity confirmed the tissue was good quality and exhibited typical morphology (Figure S12).

**Figure 6:**
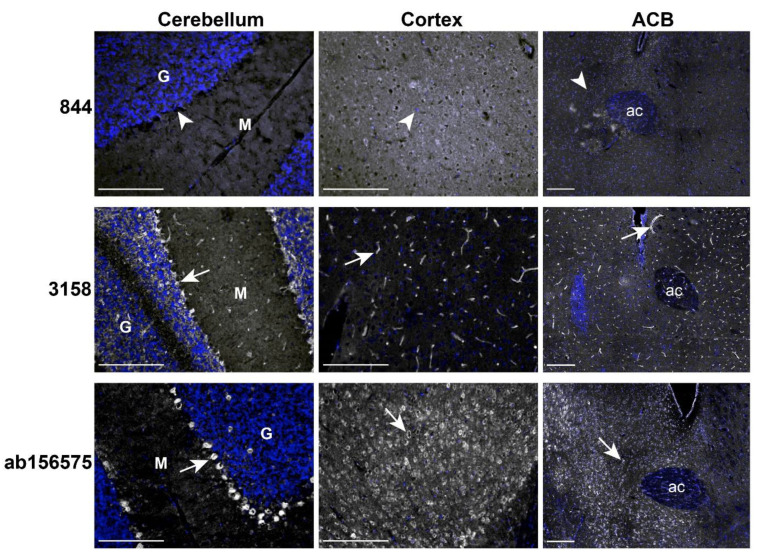
RAMP1-like immunoreactivity in mouse cerebellum, cortex and nucleus accumbens (ACB). Immunoreactivity of 844 (10 µg/mL, 3158 (1:200) and ab156575 (3.06 µg/mL) in the cerebellum, cortex, and ACB of C57BL/6J mice (NZ colony). Anti-RAMP1 immunoreactivity is shown in greyscale and nuclear staining in blue. White arrows indicate examples of positive immunoreactivity. White arrowheads indicate regions where immunoreactivity was absent. Scale bars represent 200 µm. Images are representative of immunoreactivity detected in four individual mice. HIER was performed for ab156575. G = granular, M = molecular. ac = anterior commissure.

In mouse brain, the anti-RAMP1 antibodies demonstrated remarkably different patterns of immunoreactivity to one-another in each region (Figure 6). The antibody 844 exhibited no detectable immunoreactive signal in the cerebellum, contrasting with the robust staining observed in rat (Figure 4). Similarly, no 844 immunoreactivity was present in the mouse cortex or ACB. Further experiments with heat-induced epitope retrieval and an increased concentration of the 844 antibody did not produce any clear pattern of immunoreactivity (Figures S13 and S14). The lack of 844 immunoreactivity was consistent for both male and female mice across all conditions tested. In mouse cerebellum, 3158 demonstrated a similar pattern of immunoreactivity to rat cerebellum with staining of capillary-like structures and the granular layer (Figure 6). In mouse cortex and ACB, 3158 immunoreactivity was exclusively observed in capillary-like structures and no apparent neuronal staining was present. In contrast, ab156575 produced robust immunoreactivity in mouse cerebellum between the molecular and granular layers, with weak occasional staining in the granular and molecular layers, similar to that in rat cerebellum (Figure 6). Furthermore, widespread ab156575 immunoreactivity was present in neuronal cell bodies throughout the cortex and ACB.

### 2.6. Immunofluorescent Patterns in WT and Genetic RAMP1 Mouse Model Tissue

We next profiled 844, 3158 and ab156575 in additional mouse models: (1) conditional overexpression of human RAMP1 in cells of neuronal origin (Nestin hRAMP1^cre/flox^), (2) global overexpression of human RAMP1 (global hRAMP1^WT/flox^) and (3) global RAMP1 KO (RAMP1^KO/KO^) (Table S4). This allowed us to investigate whether we could obtain any immunoreactivity for 844 in a different genetic background (129/S6-SvEv) or in C57BL/6J with human RAMP1-overexpression. These models also enabled us to examine whether patterns of immunoreactivity produced by the RAMP1 antibodies may be due to genuine RAMP1 detection, compared to off-target immunoreactivity. All three mouse models have previously been characterized and their genotype and phenotype confirmed, including quantitative PCR assessment of RAMP1 expression in the brain [34,35,36,37]. The disruption of the RAMP1 gene in the RAMP1^KO/KO^ mice is in exon 3. This model is therefore suitable for testing 844, 3158, and ab156575 which were raised against the RAMP1 C-terminal tail, which is coded downstream of this interruption (Figure S15).

**Figure 7:**
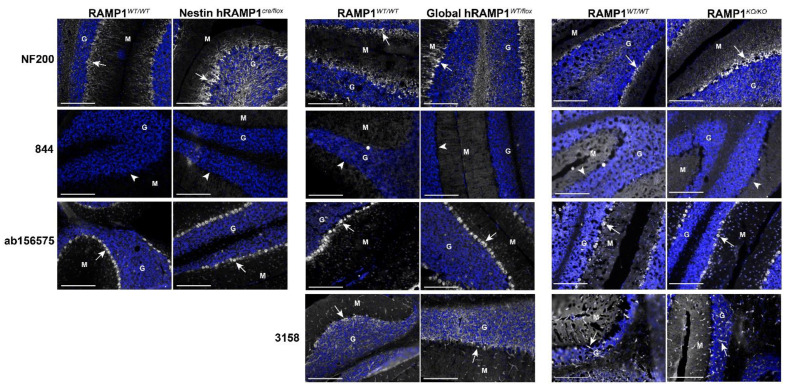
Neurofilament and RAMP1-like immunoreactivity in wildtype (WT) and hRAMP1 over-expression and knockout (KO) mouse cerebellum. Immunoreactivity of NF200 (2.72 µg/mL), 844 (10 µg/mL), ab156575 (3.06 µg/mL) and 3158 (1:200) is shown in greyscale and nuclear staining in blue. White arrows indicate examples of positive immunoreactivity. White arrowheads indicate regions where immunoreactivity was absent. Experiments with transgenic or KO mouse tissue were conducted in parallel with matched WT controls. Images are presented with the fluorescence intensity matched between the WT and transgenic or KO mouse for each antibody. Scale bar, 200 µm. Images are representative of staining detected in one (RAMP1^KO/KO^) or three individual mice (Nestin-hRAMP1 and Global-hRAMP1). HIER was performed for ab156575. G = granular, M = molecular.

Again, the cerebellum was chosen as the initial region of investigation. No differences in tissue quality or NF200 immunoreactivity were observed between the genetic models or KO mice and WT littermates (Figure 7). The antibody 844 did not demonstrate any clear immunoreactivity in the cerebellum of any of the three mouse models, even in WT controls (Figure 7). Relative to the C57BL/6J mouse (NZ colony) tissue shown in Figure 6, ab156575 and 3158 displayed very similar patterns of immunoreactivity in the cerebellum of the WT littermates of the three genetic/KO mouse models (Figure 7). When this immunoreactivity was compared to that observed in the RAMP1 overexpression models, or RAMP1 KO model, no obvious differences were observed (Figure 7). The human RAMP1 overexpression models gave an opportunity to test the apparently human-specific AF6428 antibody in mice (Figure 2 and Figure 3). However, this antibody did not produce any discernible immunoreactivity in any of the mouse models (Figure S16).

**Figure 8:**
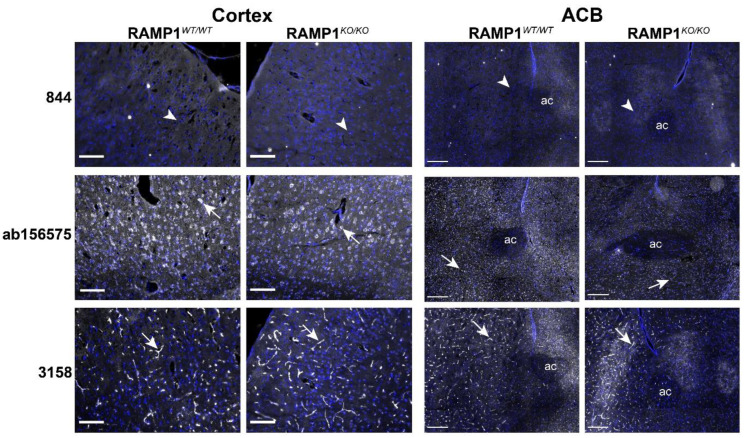
RAMP1-like immunoreactivity in WT and RAMP1 KO mouse ACB and cortex. Immunoreactivity of 844 (10 µg/mL), ab156575 (3.06 µg/mL) and 3158 (1:200) is shown in greyscale and nuclear staining in blue. White arrows indicate examples of positive immunoreactivity. White arrowheads indicate regions where immunoreactivity was absent. Scale bar, 100 µm (cortex), 200 µm (ACB). Images are representative of staining detected in three individual mice. HIER was performed for ab156575. ac = anterior commissure.

To confirm the results from KO mice, two additional regions were examined: cortex and ACB. The antibody 844 again did not exhibit any immunoreactivity in the cortex or ACB of WT or KO mice (Figure 8). ab156575 demonstrated robust immunoreactivity in cell bodies in both the cortex and ACB in both KO and WT mice. Some minor variability was observed in the pattern of immunoreactivity; cell bodies were more defined in the KO mice. However, the intensity of signal was very similar with no apparent reduction in immunoreactivity in the KO mice (Figure 8). WT and KO RAMP1 mice exhibited comparable capillary-like immunoreactivity when stained with 3158 (Figure 8). Similarly, no differences in RAMP1-like immunoreactivity were observed for the KO RAMP1 mouse with ab203282 (Figure S17).

### 2.7. Anti-RAMP1 Antibodies Detected a Range of Bands in Mouse Brain by Western Blotting

In light of the mouse tissue IHC results, we next conducted Western blots using transgenic and WT mouse tissue. We compared tissue from C57BL/6J mice (NZ colony) (corresponding to IHC shown in Figure 6) with RAMP1 KO mouse tissue and the relevant WT controls (IHC shown in Figure 7). Alongside these, transfected cell samples containing mouse RAMP1 were used as a positive control. KO and WT littermate mouse brains were divided into the cerebellum and the remaining brain regions, while C57BL/6J (NZ colony) mouse brains were divided into the cerebrum, brainstem, and cerebellum with spleen also used, enabling comparison to rat (Figure 5).

**Figure 9:**
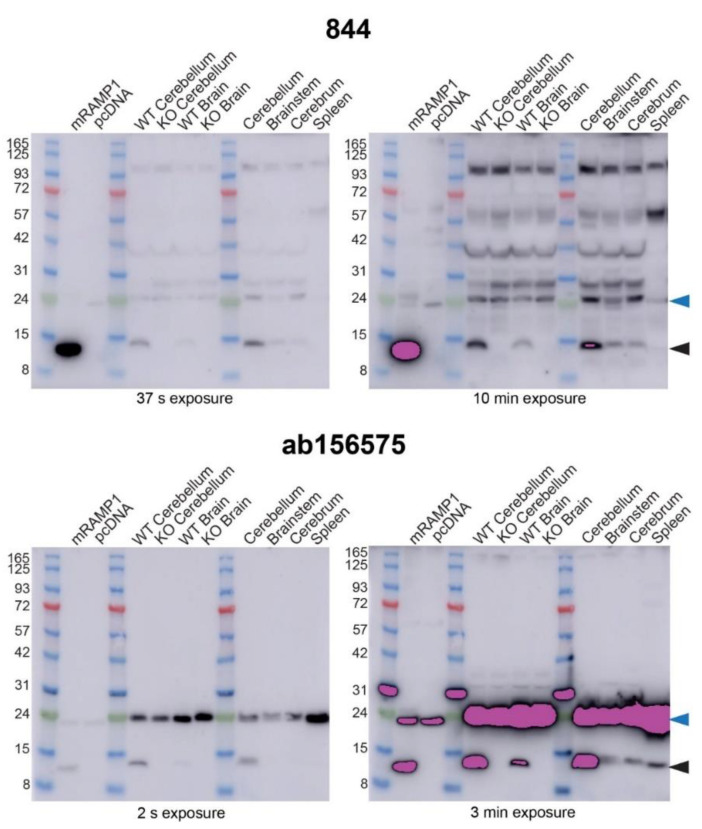
Detection of RAMP1 in mouse brain lysates by immunoblotting. Lysates were prepared from HEK293S cells transfected with mCLR:mRAMP1 or vector, or from littermate mouse WT and KO or C57BL/6J mice (NZ colony). Blots were probed with 844 (2 µg/mL) and ab156575 (0.612 µg/mL) primary antibody. Exposure times are indicated below each blot. Blots are representative of three independent experiments that used the Abcam protein ladder. Pink bands indicate signal above the range of the blot imager. Cross-reactivity is evident between a ladder protein and ab156575 in some blots. Black-filled arrowheads indicate the predicted monomeric RAMP1 band; blue-filled arrowheads mark the non-specific ~24 kDa band described in the text.

Consistent with previous Western blots, 844 detected a ~14 kDa band in transfected cell preparations expressing mRAMP1, and a band at a similar position in C57BL/6J mice (NZ colony) cerebellum, cerebrum, and brainstem (Figure 9 and Figure S18). Detection of a RAMP1-like band in the spleen samples was weak but present, as evident from the independent Western blots shown in Figure S18. The antibody 844 also produced a ~14 kDa band in samples from WT controls for the RAMP1 KO mice, but not in the RAMP1 KO mouse tissue samples (Figure 9). Similar results were obtained with ab156575; a RAMP1-like band was present at ~14 kDa (monomer) in both C57BL/6J and WT littermates but not in KO mouse brain and cerebellum samples (Figure 9). These results strongly suggest that both 844 and ab156575 can detect RAMP1 protein in mouse tissues by Western blotting (Figure 9). However, additional bands observed with both of these antibodies that were also present in KO tissue are thus likely non-specific and demonstrate that both antibodies exhibit cross-reactivity with other proteins that are not RAMP1.

## 3. Discussion

CGRP’s role in migraine and headache disorders is widely recognized. However, there is still much to learn about the contribution of each CGRP-responsive receptor to CGRP function in vivo. Mapping the anatomical localization of RAMP1, a component of both the CGRP and AMY_1_ receptors, in migraine-relevant tissues is important for CGRP-related research. Antibodies are commonly used to detect proteins in tissues. However, the major challenge is determining whether immunoreactivity can be confidently attributed to the intended protein target (specificity), or whether additional non-specific proteins are responsible, in full or in part, for the signal (selectivity). Therefore, we have profiled anti-RAMP1 antibodies with the aim of understanding the ability of each antibody to detect RAMP1, compared to its propensity to cross-react with non-specific proteins. We used a combination of transfected cell preparations and rodent tissue to test antibodies in ICC, Western blotting and IHC. Our findings are summarized in Table 1 and Table 2.

Transfected cell preparations can easily be genetically manipulated for the presence or absence of a target protein (i.e., RAMP1). Signal can therefore be correlated with the presence of the target protein, providing evidence for selectivity [21]. All anti-RAMP1 antibodies we tested could detect a band in Western blots consistent with the molecular weight of monomeric RAMP1 and most could also produce robust immunoreactivity in fixed cells transfected with RAMP1, above vector control. Our results were broadly consistent with previous examples of ICC for 844 and 3158, i.e., immunoreactivity with RAMP1 in transfected cells was previously demonstrated [23,29]. Similarly, RAMP1-like bands generated by 844 and 3158 in Western blotting have previously been observed in overexpression systems, although full blots were not shown so the possible presence of additional bands on the blot is not known [23]. In our Western blots, all antibodies produced additional bands that were likely to be non-specific, although fewer such bands were observed for ab156575. This is consistent with the improved selectivity of monoclonal antibodies, as previously demonstrated for CTR antibodies [42,43]. Some antibodies had stability issues (CRB95), had a lack of immunofluorescence in ICC (PA5-77720) or were species-specific, preventing testing in KO mouse models (AF6428). PA5-77720, which did not detect overexpressed RAMP1 in ICC in our hands, has previously been used in IHC [44]. Due to the lack of immunoreactivity in ICC, we did not test PA5-77720 in tissue; however, we cannot rule out its ability to detect RAMP1 under different conditions, and further testing is warranted. Our antibody screening does not rule out the use of these antibodies in alternative experimental contexts with sufficient controls. For example, AF6428 was used to examine and quantify RAMP1-GPCR interactions in a bead-based system [6].

While convenient, transfected cell models with overexpressed protein have limitations, such as having higher levels of expression than might be expected from tissue, where Gs-coupled GPCRs are often expressed at quite low levels because their signaling may be highly amplified [3]. We also note that we intentionally used relatively high exposure times and protein amounts to maximize the visibility of potentially cross-reactive bands. Nevertheless, the overexpression model was useful for initially screening the antibodies, followed by further validation.

The three antibodies selected for validation in rodent tissues produced distinct immunoreactive patterns in rats, yet patterns were consistent with previous reports. The antibody 3158 generated capillary-like patterns, as has been previously observed in rat brain [30]. The antibody 844 produced intense immunoreactivity between the granular and molecular layers of the cerebellum, including cell bodies and processes, which has been previously reported [14,19]. ab156575 also stained cell bodies between the molecular and granular layers, although the cells appeared more rounded than in 844 tissue preparations; this antibody has not been used for IHC in brain tissue to our knowledge. Other sites examined in the rat brain, including the LC and Sp5, also displayed considerably different immunofluorescent patterns between the 844 and ab156575 antibodies. The fiber-like immunoreactive patterns produced by 844 in the Sp5 showed some similarity to the diffuse immunoreactivity previously observed with a different anti-RAMP1 antibody [20]. However, these differed substantially from those observed with ab156575.

In mice, immunoreactive patterns were also distinctly different between antibodies. 3158 and ab156575 produced capillary-like and cell body-like immunoreactive patterns, respectively, similar to results in rat, while 844 failed to exhibit detectable immunoreactivity in mouse tissue. Immunoreactivity of 844 in fixed mouse trigeminal ganglia has been previously reported, although this immunofluorescence appeared weak [45]. Methodological differences may explain this discrepancy, although we tested multiple experimental conditions. Using tissue from mouse models with human RAMP1 overexpression also did not result in any detectable immunoreactivity with this antibody. Given the lack of immunoreactivity in these mouse models under the conditions used, we were not able to draw any conclusions about the specificity of 844 immunoreactivity in IHC, as we could not correlate the immunoreactive signal with the presence or absence of RAMP1.

Unlike 844, ab156575 did elicit immunoreactivity in mouse tissue. We would expect the immunoreactive signal to be substantially reduced in KO mice. However, there were no clear differences between genetic models/KO and WT mouse tissue. It is possible that subtle differences were present that we could not distinguish due to the limitations of IHC and imaging. Regardless, it is not possible to clearly link the immunoreactivity of ab156575 in fixed rodent tissue with the presence of RAMP1.

Our results demonstrate that multiple antibodies generated against the same protein, even using the same antigen, can produce different results. We would expect antibodies with the same target protein to produce similar immunoreactive patterns, providing support for the expression profile. This is one of the pillars of antibody validation [21]. However, this is not what we observed and the lack of consistency in patterns of immunoreactivity between anti-RAMP1 antibodies is concerning, meaning that it is extremely difficult to draw any conclusions about what immunoreactivity is genuinely driven by RAMP1 in the rodent brain.

In comparison to IHC, the Western blotting was more informative. Compared to the distinct lack of immunoreactivity in mouse brain sections, bands were observed with 844 using Western blotting of mouse tissue, as well as rat tissue. The absence of a band at the expected RAMP1 monomer molecular weight in KO mouse brain suggests that 844 can detect RAMP1, and that RAMP1 is indeed present in mouse brain. However, a wide range of additional non-specific bands was also observed. It is surprising then, that no immunofluorescence (specific or off-target) was observed in mouse brain. This may be due to differences in the epitope conformation, as Western blot samples are denatured while IHC samples are fixed. The ab156575 RAMP1 antibody produced bands in Western blot consistent with the molecular weight of RAMP1, that were absent in RAMP1 KO mice. Like 844, this provides strong evidence for the genuine detection of RAMP1 in Western blots using rodent brain preparations. Similar results have previously been seen in Western blots using this antibody and brainstem tissue from RAMP1 KO mice [46], consistent with our results. However, ab156575 also produced a recurring additional band at ~24 kDa. This band has previously been reported, although it has been reported as both a non-specific band [47] and a monomeric RAMP1 band [48,49]. Given our comparisons between gel running conditions, and between species of RAMP1 in transfected cells that allowed us to separate RAMP1 monomers from dimers, it seems unlikely that this can be monomeric RAMP1. Furthermore, given that this band was present in both WT and KO mouse samples, and that mass spectrometry did not detect RAMP1 in samples from this region of the gel, we suggest that this band is due to off-target binding. We suggest that ab156575 could be used in carefully considered Western blotting experiments to detect RAMP1, while recognizing the limitation of the non-specific ~24 kDa band, which reduces the ability of the antibody to display potential RAMP1 dimeric bands clearly. The non-specific band may be present in some tissues but not in others.

A clear challenge with interpreting the current and previous work with these RAMP1 antibodies is that they detect both RAMP1 and other protein(s). The antibodies 844 and 3158 have been used in many studies identifying the protein localization of RAMP1 in nervous tissue (Table S1) but our results also have implications for the interpretation of results generated using other antibodies. IHC results may be heavily influenced by false-positives, especially if the non-RAMP1 protein detected has a greater abundance than RAMP1 itself. The RAMP1 signal would then be masked. This may be the case for ab156575 immunoreactivity in the cerebellum. Some proteins detected by mass spectrometry in the non-specific band region are reported to be expressed in the cerebellum, such as peroxiredoxin-2 and 40S and 60S ribosomal protein subtypes. These proteins could potentially contribute to ab156575 immunoreactivity (Table S3) [50,51]. A BLAST search using the 844 antigenic sequence provided a range of potentially cross-reactive proteins with sequence similarity, some of which overlap with the proteins observed in the non-specific mass spectrometry band and could be investigated further (Table S3). We also note that the pattern of 844 immunofluorescence could be similar to a cytoskeletal protein (e.g., NF200) and an interaction between RAMP1 and beta-tubulin has previously been observed [52].

Western blotting shows that ab156575 and 844 can detect RAMP1 (as well as other proteins) in brain tissue samples but immunoreactivity patterns in brain with these antibodies do not correlate with one-another. What then can we conclude about RAMP1 expression? Radiolabeled CGRP binds intensely to the cerebellum, ACB, LC, and AP, suggesting the presence of RAMP1 as a key subunit of CGRP receptors [9,10,12]. RAMP1 mRNA probes support the presence of RAMP1 in these regions, along with the cortex which shows weak CGRP binding but abundant RAMP1 mRNA in some layers [13,15]. The Sp5/sp5 shows moderate CGRP binding and RAMP1 mRNA, while the reticular nucleus is relatively free of either signal [13,28,29].

Identifying likely RAMP1 expression within tissue substructures or specific cells based on ligand binding and mRNA probe studies is challenging. These studies give a good indication of which brain regions, and their broad subregions or layers may express RAMP1. However, due to the constraints of the techniques at the time, they are relatively low resolution and do not allow localization at the cellular level. More recent studies using small molecule binding (MK-4232) detected using positron emission tomography (PET) can still only provide low-resolution information, such as describing binding in the cerebellum, but not the specific layer [53]. Therefore, immunoreactivity should be an ideal candidate to elucidate the expression of RAMP1 in higher resolution. Our initial results in rat cerebellum with 844 and ab156575 suggest that the IHC immunoreactivity broadly aligns with the binding, mRNA and PET studies. However, closer inspection of immunoreactivity with these antibodies shows a lack of consistency with mRNA and binding data. For example, we observed 3158 immunoreactivity in the granular layer. This contrasts with previous reports, which note the absence of binding and mRNA detection in this layer of the cerebellum [9,10,14,15]. Similarly, our study found a lack of immunoreactivity in the LC with 844, contrary to the strong binding previously observed [10,28]. Overall, this highlights that careful comparison with mRNA and binding data is required to interpret IHC. It also demonstrates that relying solely on immunoreactivity to localize RAMP1, or any protein expression, is inadvisable.

Although the antibodies tested in this study did not display immunoreactivity that we can confidently attribute to RAMP1, there may be other antibodies, such as other commercially available antibodies or dual-target antibodies which detect CLR:RAMP1, that could be used to identify the spatial distribution of RAMP1. For example, erenumab is reported to bind to the molecular layer of human and cynomolgus monkey cerebellum [54]. The tool antibody AA32, raised against CLR:RAMP1, generated a pattern of binding in laminae I-III of the Sp5 and the cerebellum, which is broadly consistent with binding and mRNA data [28,29,55]. However, these dual-target antibodies may not be commercially available and it is not yet clear whether these antibodies exclusively bind to CLR:RAMP1 or also bind to CTR:RAMP1 [56,57]. Furthermore, these antibodies are reported to be highly selective for primate, over rodent, making studies in genetically modified mouse models difficult [58].

Further investigation into RAMP1 expression could leverage alternative approaches rather than antibodies. For example, transgenic mouse models with labelled proteins or optimized mass spectrometry could be used [33]. While localizing mRNA can only suggest the presence of functional protein, not confirm it, modern sophisticated RNA-based approaches such as spatial fluorescence in-situ hybridization or single-cell sequencing are increasingly used and can provide evidence for expression at a cellular resolution [36,59,60]. Similarly, pharmacological and functional studies using specific ligands can also help tease apart the contributions of each CGRP family receptor to migraine biology [2].

The goal of this research was to identify RAMP1 antibodies that could be used to identify the molecular correlates of radiolabeled ligand/CGRP binding sites. Further tools and strategies will be needed to work towards this goal, given that the antibodies we have profiled in this study each produce different patterns of immunoreactivity that cannot be clearly linked to RAMP1. We need to revisit the structures where we think RAMP1 is expressed and confirm these studies with additional data.

## 4. Materials and Methods

### 4.1. Antibodies

Seven anti-RAMP1 antibodies were used, as detailed in Table S2. For blocking peptide experiments, a peptide corresponding to the human RAMP1 C-terminal sequence was synthesized in-house (Figures S19–S21) and primary antibodies (844 and ab156575) were pre-incubated with 50 µM peptide for 1 h at 20 °C. The primary antibody antigens, where known, and species-specific sequence information, are shown in Figure 1 and Figure S15. The secondary antibodies used are detailed in Table S5. An anti-myc or anti-FLAG tag antibody was used as a control for some experiments (Figure S2).

### 4.2. Animals and Tissue Collection

All procedures involving the use of rodents and their care were approved and conducted in accordance with the guidelines described in the Supplemental Information, including ARRIVE2 guidelines [61]. Additional information is provided in the Supplemental Information, including a summary in Table S4.

### 4.3. Rodent Tissue Sourced within New Zealand

Male and female Sprague Dawley (SD) rat and C57BL/6J mouse brains were obtained from the Integrated Physiology Unit (University of Auckland, Auckland, New Zealand). All procedures involving the use of animals were conducted in accordance with the New Zealand Animal Welfare Act (1999) and approved by the University of Auckland Animal Ethics Committee. Rodents of the same sex were housed with littermates in Techniplast Greenline IVC with Sealsafe Plus GM500 cages (mice) or as pairs in Techniplast Conventional 1500U cages (rats) in a controlled environment (12 h light–dark cycle; room temperature, 22 ± 2 °C) with ad libitum access to standard chow (Teklad TB 2018; Harlan, Madison, WI, USA) and water. Cages also contained an additional enrichment item (house or toy). Tissue was collected from animals culled as part of routine colony maintenance. The estrous cycle phase was not assessed or recorded for female rats or mice. Anesthesia was induced with 5% isoflurane in 2 L/min O_2_, and the animals killed by cervical dislocation. Brains (dissected into cerebellum, brainstem and cerebrum) and spleen for Western blotting were snap-frozen in liquid nitrogen and stored at −80 °C. Brains for immunohistochemistry were fixed by immersion in 4% paraformaldehyde (PFA) post-dissection (4–21 h). After fixation, tissues were cryoprotected with 20% sucrose (*w*/*v*) in phosphate-buffered saline (PBS) and embedded in optimal cutting temperature compound (Sakura Tissue-Tek, 4583). Brains were cryo-sectioned coronally at a thickness of 10 µm using a Leica CM1850 microtome (Leica Biosystems, Wetzlar, Germany). Sections were mounted onto slides and then stored at −80 °C.

### 4.4. Genetic Mouse Model Tissue

RAMP1 (KO) mouse brains were generously provided by the Caron laboratory or the Lutz laboratory and were generated as previously described [37,62]. Although provided by two different groups, this was the same mouse line that originated in the Caron laboratory. Brains from global-hRAMP1 or nestin-hRAMP1 overexpression mice were generously provided by the Russo laboratory and were generated as previously described [34,35]. Details on the background, origin, sex, weight, age and tissue preparation of the control and genetic mouse models are provided in Table S4.

### 4.5. Cell Culture and Transfection

HEK293S cells were cultured, transfected and plated as previously described [43,63]. The N-terminally tagged myc-RAMP1, FLAG- or untagged human RAMP1, N-terminally FLAG-tagged human RAMP2, N-terminally tagged myc human RAMP3, N-terminally HA-tagged human CLR and CTR constructs in pcDNA3.1 were as previously described [24,64,65,66]. Unmodified pcDNA3.1 construct was used as a vector control. Untagged rat CLR, CTR and RAMP1 constructs in pCMV6 plasmids were obtained from Origene (Rockville, MD, USA). Untagged mouse CLR, CTR and RAMP1 in pCMV6 plasmids were as previously described [67]. RAMP1 was transfected in a 1:1 ratio with CLR or CTR. The CTR splice variant was CT_(a)_ for all species. Vector-transfected control cells were transfected with pcDNA3.1 alone. In our hands, vector or CLR-transfected HEK293S cells have little to no functional response (cAMP) to CGRP, suggesting minimal or no expression of RAMP1 [66].

### 4.6. Western Blotting

Protein preparations for Western blotting from transfected HEK293S cells and tissues are described in the Supplemental Information. Protein samples (1–100 µg, Table S6) were loaded alongside the Benchmark (Cat# 10748010, Life Technologies, Carlsbad, CA, USA), PageRuler (Cat# 26616, Thermofisher, Waltham, MA, USA) or ab116027 (Abcam, Cambridge, UK) protein ladders onto 4–12% SurePage Bis-Tris gels (GenScript, Piscataway, NJ, USA). Western blotting was performed as previously described [43]. Under some conditions, 2-(N-morpholino)ethanesulfonic acid (MES) running buffer was used to aid in the separation of lower molecular weight bands. Figure legends provide detailed information about the conditions used.

### 4.7. ICC and IHC

Transfected HEK293S cells were fixed with 4% PFA, washed with TBS containing 0.1% Tween20 (TBS-T) and ICC performed as previously described [43]. Rodent brain sections were processed for IHC as previously described [4,43]. Antigen retrieval was performed for ab156575 in rodent IHC, as stated in relevant figure legends. Antigen retrieval involved incubation of sections with 10 mM Tris-sodium citrate buffer containing 0.05% Tween20 with brief microwave heating. For brains from control and genetically modified mice, IHC was performed in a paired experimental design.

### 4.8. Image Preparation and Analysis

ICC and IHC images were acquired using an Operetta (PerkinElmer, Waltham, MA, USA) high-content imager with a 20× objective and processed as detailed in the Supplemental Information. Images were minimally processed using ImageJ to adjust color and brightness for presentation purposes. Any processing was uniformly applied across each image, and where appropriate, across all conditions for an antibody. For interpreting IHC signal from WT littermate and genetic mouse model tissue, images were compared with identical processing, including matched fluorescence intensity. Representative ICC, Western blotting and IHC images are presented from independent experiments performed using separate antibody dilutions. ICC-independent experiments are defined as the immunoreactivity detected in cells from independent transfection and ICC experiments, performed with two technical replicates. Independent experiments for Western blotting are defined as individual preparations from transfected cells, or lysates prepared from individual rodents. IHC-independent experiments are defined as immunoreactivity detected in brain sections from individual rodents. Figure legends describe the degree of experimental replication. Due to tissue availability of RAMP1 KO mouse cerebellum, only pilot experiments in one individual mouse were performed.

## 5. Conclusions

In summary, all antibodies could detect RAMP1 in transfected cells. However, all antibodies also produced off-target binding to some degree. Under the conditions we tested, we cannot recommend the use of any of the RAMP1 antibodies we tested for IHC; however, ab156575 may be useful for specific Western blotting applications. We highlight in this study the challenges of confirming the genuine detection of RAMP1 protein using antibodies. This has implications for our current understanding of the expression of RAMP1, which may not be as clear-cut as currently thought. This is important for CGRP family receptors as migraine targets, but also other GPCRs which interact with RAMP1. The validation framework we present may be informative to others in characterizing and understanding the limitations of these and other RAMP1 antibodies. This study also highlights the need for thorough validation, using multiple methods, for antibodies as tools in IHC and other methods, not only for RAMP1 but for other proteins. The work also emphasizes the importance of using multiple antibodies against a protein target to either increase confidence in results or reveal possible discrepancies. Further work should revisit RAMP1 expression using optimized, well-validated tools.

## Figures and Tables

**Table 1:** Summary of major bands detected with anti-RAMP1 antibodies in Western blots of samples from transiently transfected HEK293S cells under the conditions tested.

Antibody	RAMP1 Band (~14 kDa) [Black]	Dimeric RAMP1 Band [Red]	Additional ~24 kDa Band (Non-Specific) [Blue] ^&^
*844*	human, rat, mouse	human ^^^, rat *	No
*3158*	human, rat, mouse	human ^^^	Yes
*ab156575*	human, rat, mouse	human ^^^, rat *	Yes
*ab203282*	rat, mouse	No	Yes
*AF6428*	human	No	No
*PA5-77720*	rat, mouse	No	Yes
*CRB95* ^†^	human	human ^^^	No

* band was ~24 kDa, ^^^ band was ~25–28 kDa. ^&^ all tested antibodies produced additional non-specific bands. ^†^ CRB95 was only tested against human RAMP1. Colors refer to arrowheads in certain figures used to indicate bands. This table summarizes the major bands discussed in the text.

**Table 2:** Summary of antibody validation.

Antibody	ICC	Rat		Mouse		Mouse Overexpression	Mouse KO	
		IHC	WB ^#^	IHC	WB ^#^	IHC	IHC	WB ^#^
*844*	Detected h/r/m RAMP1	Between Gran/Mol layers (Cb)	Cb, BS, Cortex, Spleen	No IR	Cb, BS, Cortex, Spleen	No IR, no difference to WT	No IR, no difference to WT	RAMP1 monomer absent in KO
*3158*	Detected h/r/m RAMP1	Granular layer (Cb)	NP	Cb, Cortex, ACB	NP	NP	No difference to WT	NP
*ab156575*	Detected h/r/m RAMP1	Between Gran/Mol layers (Cb)	Cb, BS, Cortex, Spleen	Nuclei/cell bodies in Cb, Cortex and ACB	Cb, BS, Cortex, Spleen	No difference to WT	No difference to WT	RAMP1 monomer absent in KO
*ab203282*	Detected h/r RAMP1	NP	NP	NP	NP	NP	No difference to WT	NP
*AF6428*	Detected hRAMP1 ^	NP	NP	NP	NP	No IR, no difference to WT	NP	NP
*PA5-77720*	Did not detect RAMP1	NP	NP	NP	NP	NP	NP	NP
*CRB95*	Detected h/r RAMP1 *	Between Gran/Mol layers (Cb)	NP	NP	NP	NP	NP	NP

^ Highly variable between independent experiments and high non-specific immunoreactivity in all conditions. * mouse not examined. ^#^ refers to RAMP1 monomer. ACB, nucleus accumbens; BS, brainstem; Cb, cerebellum; Gran, granular; h, human; IR, immunoreactivity; KO, knockout; Mol, molecular; m, mouse; NP, not performed; r, rat; WT, wildtype.

## Data Availability

The data generated for the current study not available in the main text or supplemental information are available from the corresponding author on reasonable request.

## Supplementary Materials

The supporting information can be downloaded at: https://www.mdpi.com/article/10.3390/ijms232416035/s1. References [68,69,70,71,72,73,74,75,76,77,78,79,80,81,82,83,84,85,86,87,88] are cited in the supplementary materials.
