# Supplementary material for: Characterization of Antibodies against Receptor Activity-Modifying Protein 1 (RAMP1): A Cautionary Tale
Source: Int J Mol Sci. 2022 Dec 16;23(24):16035. doi: 10.3390/ijms232416035 (PMC9787598; doi:10.3390/ijms232416035)
Supplement: Supplementary file 1 [file ijms-23-16035-s001.zip › ijms-2055689-supplementary.pdf]

Supplemental Information

Characterization of antibodies against receptor activity-modifying protein 1 (RAMP1): a cautionary tale

Erica R Hendrikse[1^], Tayla A Rees[1^], Zoe Tasma[1], Michael L Garelja[2], Andrew Siow[1,3], Paul WR Harris[1,3,4], John B Pawlak[5], Kathleen M Caron[5], Elizabeth S Blakeney[5], Andrew F Russo[6,7], Levi P Sowers[6,7], Thomas A Lutz[8], Christelle Le Foll[8], Christopher S Walker[1,4], Debbie L Hay[2,4*]

[1]School of Biological Sciences, The University of Auckland, Auckland, New Zealand

[2]Department of Pharmacology and Toxicology, The University of Otago, Dunedin, New Zealand

[3]School of Chemical Sciences, The University of Auckland, Auckland, New Zealand

[4]Maurice Wilkins Centre for Molecular Biodiscovery, The University of Auckland, Auckland, New Zealand

[5]Department of Cell Biology and Physiology, University of North Carolina at Chapel Hill, Chapel Hill, North Carolina, USA

[6]Department of Molecular Physiology and Biophysics, University of Iowa, Iowa City, Iowa, USA

[7]Center for the Prevention and Treatment of Visual Loss, Veterans Administration Health Center, Iowa City, IA, USA 52246.

[8]Institute of Veterinary Physiology, University of Zurich, Zurich, Switzerland

^Authors contributed equally

*Correspondence: Debbie L Hay, debbie.hay@otago.ac.nz

Supplemental Methods

Rodent origins and care

All procedures involving the use of rodents and their care were conducted in accordance with the New Zealand Animal Welfare Act (1999) and approved by the University of Auckland Animal Ethics Committee (rats and C57BL/6J mice) (Ghanizada et al., 2021), with the EU Directive on the protection of animals used for scientific purposes and approved by the Veterinary Office of the Canton Zurich, Switzerland (RAMP1 knockout (KO) and wild-type (WT) mice) (Coester et al., 2020), approved by the Institutional Animal Care and Use Committee of the University of North Carolina-Chapel Hill and the Institutional Animal Care and Use Committee of the University of California San Francisco (Li et al., 2014), or approved by the University of Iowa Animal Care and Use Committee and performed in accordance with the standards set by the National Institutes of Health and the ARRIVE guidelines (overexpression and WT mice) (Bohn et al., 2017; Zhang et al., 2007).

The parental hRAMP1 transgenic mice have an expression cassette containing a floxed GFP stop sequence upstream of the hRAMP1 allele. Upon cre-dependent removal of the stop sequence, hRAMP1 expression was driven by the CX1 promotor (CMV enhancer and chicken β-actin promoter). Global overexpression hRAMP1 mice were generated from germline removal of the GFP stop sequence by a cross with EIIA-cre mice followed by backcrosses with non-transgenic littermates, as previously described (Bohn et al., 2017). The nestin-hRAMP1 transgenic mice were generated by a cross with nestin-cre mice to allow conditional expression of hRAMP1 in nervous tissue. In brain tissues, these mice have ~2x the total amount of RAMP1 mRNA compared to WT mice, comprising mRAMP1 and hRAMP1(Zhang et al., 2007). Tissues were taken from 10-20 week old mice.

Male WT and RAMP1 KO (18 week-old) were sacrificed after 2h fasting prior to dark onset. At dark onset, mice were anesthetized with pentobarbital (100 mg/kg, Kantonsapotheke Zurich, Switzerland). After reaching deep anesthesia, mice received cardiac perfusion for 1.5 min with 0.1 M phosphate buffer (PB) followed by 2.5 min of 4% paraformaldehyde in 0.1 M PB (PB-PFA, pH 7.4). Brains were postfixed for 4 h in 2% PB-PFA and cryoprotected in 20% sucrose in 0.1 M PB for 24 h. Cryoprotected brains were subsequently frozen in hexane on dry ice and stored at -80 °C. Another cohort of male mice (28 week-old) was decapitated and their brain frozen on dry ice until further analysis.

Western blotting

Transfected HEK293S cells in 150 mm dishes were chilled on ice and washed in ice-cold PBS. Fresh PBS was added to harvest cells using a cell scraper. For initial antibody characterization (Figure 2) membrane-enriched protein preparations were used. Cells were pelleted at 4000 x *g* for 10 minutes at 4°C and the supernatant discarded. Cell pellets were resuspended in 2 mL solubilization buffer (Tris-NaCl, pH 8.0) containing a complete mini EDTA-free protease inhibitor cocktail (1:10,000, Roche

Applied Science, Germany) and lysed by sonication (Qsonica Q700, amplitude 10, pulse 1 second on/off for 1 minute). Samples were centrifuged (30,000 x *g* for 30 minutes at 4°C) and the supernatant discarded. Pellets were resuspended in solubilization buffer and 1% n-dodecyl-β-D-maltopyranoside (DDM)/0.1% Cholestryl hemisuccinate tris salt (CHS) and left to solubilize for 2 hours on an end-over-end mixer at 4°C. Insoluble material was removed by centrifugation (30,000 x *g* for 30 minutes at 4°C) and the supernatant aliquotted and stored in protein Lo-bind tubes (Eppendorf, Germany) at -80°C until use. Each aliquot was freeze-thawed a maximum of three times.

To closely match the rodent tissue preparations and serve as a more appropriate control, transfected HEK293S cells were also prepared as lysates (Figures 5 and 9). For preparation of lysate samples, transfected HEK293S, fresh-frozen mouse or rat brain tissue and spleen (< 100 mg) were homogenized using a 1 mL Dounce glass homogenizer in RIPA buffer (Tris-NaCl, 0.1% SDS, 0.5% sodium deoxycholate, 1% Triton X-100, pH 8.0) containing a complete mini EDTA-free protease inhibitor cocktail (1:10,000; Roche Applied Science, Germany). The homogenized tissue was then left to solubilize on an end-over-end mixer for 2 hours at 4°C. Samples were pelleted by centrifugation (16,000 x *g*, 20 minutes, 4°C) and the supernatant aliquoted into protein Lo-bind tubes and stored at -80°C.

Protein concentrations of membrane-enriched or tissue lysates were quantified using a BCA protein assay kit, using buffers matched to the preparation of the lysate samples (ThermoFisher Scientific, MA, USA).

Protein samples were incubated for 1 hour at 37°C in 4x loading dye (2.5 mL 1M Tris-HCL, 4 mL 20% sodium dodecyl sulfate, 4 mL 100% glycerol, 0.04 mg bromophenol blue) and 0.1M ddithiothreitol (DTT). Protein was loaded alongside a protein ladder onto 4-12% SurePage SDS gels and run at 180 V in MOPS or MES buffer. Proteins were transferred to 0.45 μm PVDF membranes (Life Technologies, CA, USA) and were blocked with 5% non-fat milk in TBS-T for 1 hour at room temperature. Blots were incubated with primary antibody overnight at 4°C, washed in TBS-T then incubated with secondary antibody (1:2,000 or 1:10,000) for 1 hour at room temperature. Blots were developed with Supersignal West Pico Plus ECL (ThermoFisher Scientific) and imaged using an Amersham Imager A600 (GE Healthcare, IL. USA).

Image acquisition and adjustment

ICC and IHC images were primarily imaged using an Operetta high-content imager (PerkinElmer) with a 20x high numerical aperture (NA) objective lens (0.75 NA) or a 40x long working distance objective lens (0.6 NA). Some IHC images were acquired using the Vectra Polaris (Akoya Biosciences) with a 20x (0.5 μm/pixel) objective lens. Images were acquired from the Harmony 4.1 software (Operetta) or Phenochart software (Polaris) as 16-bit TIFF files. When required, images were stitched using the grid/collection FIJI algorithm and 5-20% overlap (Preibisch et al., 2009). Using ImageJ, the images

were then pseudo-coloured to greyscale (immunoreactivity) and blue (nuclei), adjusted for colour and brightness for presentation purposes and a scale bar added. Any adjustment was applied uniformly across all images for an antibody. Care was taken to avoid misrepresentation or loss of data, such as losing the darkest or brightest pixels in a given image by clipping the dynamic range (Johnson, 2012). Staining was defined as detectable when it could be visually discerned above background compared to controls under identical imaging conditions, specifically 'no primary antibody' controls (not shown). Image panels were prepared in Adobe Illustrator. Brain region nomenclature is consistent with the Allen Mouse Brain Atlas (Lein et al., 2007).

Mass spectrometry (LC-MS/MS) analysis

For sample preparation, 50 µg rat CLR:RAMP1 HEK293S lysate (to investigate the 14 and 24 kDa bands), 100 µg of rat spleen or cerebellum (to investigate the 14 kDa band), or 80 µg rat cerebellum or brainstem (to investigate the 24 kDa band) were loaded into a 4-12% gel and run using MES buffer alongside the Abcam protein ladder. Using the ladder as a reference, bands were cut from the unstained gel between the 8-15 kDa markers (~14kDa band) and 24 kDa marker, corresponding to the non-specific band region identified by some antibodies, including ab156575. Gel bands were washed in milliQ water and stored at -20 °C until required.

Gel bands were dehydrated with acetonitrile, then subjected to reduction with DTT, alkylation with iodoacetamide, and digestion with 12.5 ng/µL porcine trypsin (Promega, WI, USA) in a temperature-controlled microwave (CEM, NC, USA) at 45°C for 1 hour. The digest was acidified and injected onto a 0.3x 10 mm trap column packed with Reprosil C18 media (Dr Maisch, Germany) and desalted for 5 minutes at 10µL/min before being separated on a 0.075 x 200 mm picofrit column (New Objective, MA, USA) packed in-house with 3u Reprosil C18 media. The following gradient was applied at 300nL/min using a NanoLC 400 UPLC system (Eksigent, CA, USA): 0 min 1%B; 0.1 min 5%B; 16 min 40%B; 17.5 min 98%B; 19.5 min 98%B; 20 min 1%B; 30 min 1%B, where A was 0.1% formic acid in water and B was 0.1% formic acid in acetonitrile.

The picofrit spray was directed into a TripleTOF 6600 Quadrupole-Time-of-Flight mass spectrometer (Sciex, Canada) scanning from 300-2000 m/z for 200 ms, followed by 40 ms MS/MS scans on the 35 most abundant multiply-charged peptides (m/z 80-1600). The mass spectrometer and HPLC system were under the control of the Analyst TF 1.8 software package (Sciex).

For the monomer (14 kDa) and predicted dimer (24 kDa) samples from HEK293S cells transfected with rat RAMP1, resulting MS/MS data were searched against an in-house database comprising rat protein sequences from Uniprot.org plus a set of common contaminant sequences (30,106 entries in total), using ProteinPilot version 5.0 (Sciex). Search parameters were as follows: Sample Type, Identification; Cys Alkylation, Iodoacetamide; Digestion, Trypsin; Special factor, Gel band; Search Effort, Thorough.

To determine what the 24 kDa non-specific band detected by anti-RAMP1 antibodies in human-derived HEK293S cells could be, the resulting MS/MS data were searched against an in-house database comprising human protein sequences from Uniprot.org plus a set of common contaminant sequences (78,747 entries in total), using ProteinPilot version 5.0 (Sciex). Search parameters were as follows: Sample Type, Identification; Cys Alkylation, Iodoacetamide; Digestion, Trypsin; Special factor, Gel band; Search Effort, Thorough; ID Focus, Biological Modifications and Amino Acid Substitutions.

For rat tissue samples of the 14 kDa monomer band (spleen) and 24 kDa non-specific band (cerebellum and brainstem), resulting MS/MS data were searched against an in-house database comprising rat protein sequences from Uniprot.org plus a set of common contaminant sequences (30,106 entries in total), using ProteinPilot version 5.0 (Sciex). Search parameters were as follows: Sample Type, Identification; Cys Alkylation, Iodoacetamide; Digestion, Trypsin; Special factor, Gel band; Search Effort, Thorough.

The peptide summaries exported from ProteinPilot were further processed in Excel using a custom macro to remove proteins with Unused Scores below 1.3, eliminate inferior or redundant peptide spectral matches, and to sum the intensities for all unique peptides from each protein.

Supplementary Tables

Table S1. Summary of previous IHC and western blotting data in nervous tissue for RAMP1 antibodies profiled in this study

Ab code, origin	Ab type		Regions examined	References
844, Merck/CRB	Goat pAb	Mouse	TG	(Vilotti et al., 2016)
		Other species	Brain and PNS regions in rat, monkey, and human	(Csati et al., 2012; Edvinsson et al., 2019; Edvinsson et al., 2011; Edvinsson et al., 2020; Eftekhari & Edvinsson, 2011; Eftekhari et al., 2010; Eftekhari, Salvatore, et al., 2013; Eftekhari et al., 2015; Eftekhari, Warfvinge, et al., 2013; Filiz et al., 2019; Pozo-Rosich et al., 2015; Summ et al., 2010; Warfvinge & Edvinsson, 2019; Warfvinge et al., 2020)
Ab156575, Abcam	Rabbit mAb	Mouse	Schwann cells, ventral tegmental area, laterodorsal tegmental nucleus, nucleus accumbens	(Aranas et al., 2021; De Logu et al., 2022)
		Other species	Human Schwann cells, rat brainstem and hypothalamus	(De Logu et al., 2022; Ernstsen et al., 2022; Kang et al., 2019)
3158, Merck/CRB	Rabbit pAb	Mouse	None	None
		Other species	Rat cerebellum, Sp5, TG, spinal cord, retinal ganglion cells	(Blixt et al., 2017; Edvinsson et al., 2011; Eftekhari & Edvinsson, 2011; Eftekhari et al., 2010; Eftekhari, Warfvinge, et al., 2013; Seiler et al., 2013; Sheykhzade et al., 2017; Walker et al., 2015)
PA5-77720, ThermoFisher	Rabbit pAb	Mouse	TG and Sp5	(Moye et al., 2021)
		Other species	None	None
ab203282, Abcam	Rabbit pAb	Mouse	Brain	(Xu et al., 2021)
		Other species	Rat spinal cord	(Zhou et al., 2017)

pAb, polyclonal, mAb, monoclonal. Sp5, spinal trigeminal nucleus; TG, trigeminal ganglia. Note: relatively few studies have used RAMP1 antibodies to examine RAMP1 expression in mouse nervous tissues, hence this has been highlighted in our table.

Table S2. Primary antibodies

	Host, isotype, Concentration	Antigen, Purification method	Source, Identifier (Cat#, Clone, Lot, RRID)
**Anti-RAMP1 antibodies**			
844	Goat pAb, IgG ~1 mg/mL	C-terminal hRAMP1 antigen Ahx-QSKRTEGIV Affinity purification	Non-commercial antibody produced for Merck by Cambridge Research Antibodies (Eftekhari et al., 2010) No RRID
3158	Rabbit pAb IgG Unknown mg/mL	C-terminal rRAMP1 antigen Ahx-RSKRTEGIV Affinity purification	Non-commercial antibody produced for Merck by Cambridge Research Antibodies (Eftekhari et al., 2010) No RRID
ab156575	Rabbit mAb IgG 0.612 mg/mL	C-terminal hRAMP1 antigen between amino acids 100-148 Protein A purification	Abcam, ab156575, Clone EPR10867, Lot GR31964031 RRID: AB_2801501
ab203282	Rabbit pAb IgG 1 mg/mL	C-terminal mRAMP1 antigen between amino acids 100-148 Protein A purification	Abcam, ab203282, Lot GR3320406 No RRID
AF6428	Sheep pAb IgG 0.2 mg/mL	N-terminal hRAMP1 antigen between amino acids 27-117 Affinity purification	R&D, AF6428, Lot CBSD0116081 RRID: AB_10718851
PA5-77720	Rabbit pAb IgG 0.85 mg/mL	N-terminal rRAMP1 antigen between amino acids 27-38 Affinity purification	Invitrogen, PA5-77720, Lot WJ3394252 RRID: AB_2736495
CRB95	Rabbit pAb IgG 0.4 mg/mL	C-terminal hRAMP1 antigen Ahx-QSKRTEGIV-acid Affinity purification	Discovery Antibodies (Cambridge Research Biochemicals), crb2005038 RRID: AB_2801502
**Anti-tag antibodies**			
anti-myc	Mouse mAb IgG1 0.2 mg/mL	C-myc tag, EQKLISEEDL Protein G purification	Merck, OP10, Clone 9E10, Lot D00123355 RRID: AB_10682957
anti-FLAG	Mouse mAb IgG1 1 mg/mL	FLAG tag, DYKDDDDK Affinity tag purification	Sigma-Aldrich, F1804, Lot SLBV9325 RRID: AB_2827968
**Anti-marker antibody**			
Anti-NF200	Rabbit pAb IgG 0.543 mg/ml	Human neurofilament heavy amino acids 1-100 Protein A purification	Abcam, ab207176, Clone EPR20020, Lot GR284807, RRID AB_2827968

Table S3. Proteins detected by mass spectrometry from the ~24 kDa non-specific band in HEK293S cells and rat cerebellum. Proteins are listed in order of their abundance as detected in mass spectrometry.

**Name**	**MW (kDa)**	**BLAST***	**Expression in cerebellum+**
Peroxiredoxin-2	22	N	Purkinje cells and molecular layer
40S ribosomal protein S11	18	Y	No expression
60S ribosomal protein L26	17	Y	Purkinje cells and molecular layer
40S ribosomal protein S5	25	N	Purkinje cells
Transgelin-2	22	Y	Low expression
Peroxiredoxin-1	19	N	Granular and molecular layers
Phosphatidylethanolamine-binding protein 1	21	N	Purkinje cells and granular layer
Peptidyl-prolyl cis-trans isomerase B	20	N	Purkinje cells
60S ribosomal protein L23a	18	N	No expression

*Proteins detected by mass spectrometry were also detected in the top 20 results from a protein BLAST search of the 844 antigenic sequence (shown in Figure 1) against the non-redundant protein database.

+Expression data taken from The Human Protein Atlas (Uhlen et al., 2015).

Table S4. Details of the rats and mice used for each figure.

Figure	Animal	Origin	Details (Sex, weight, age)	Tissue preparation
4 (IHC)	SD rats*	Auckland, NZ	Male and female, 272-609 (g), 14-24 weeks	4% PFA immersion fixed 4-21 hours. Cryoprotected in 20% sucrose and embedded in OCT. Sectioned at 10 µm.
5 (WB)	SD rats*	Auckland, NZ	Male and female, 258-713 (g), 14-23 weeks	Fresh-frozen in liquid nitrogen. Brains dissected into cerebellum, brainstem, and cerebrum for tissue lysate for western blotting.
6 (IHC)	C57BL/6J mice^	Auckland, NZ	Male and female, 21.8-27.6 (g), 11-14 weeks	4% PFA immersion fixed 24 hours. Cryoprotected in 20% sucrose and embedded in OCT. Sectioned at 10 µm.
7 (IHC)	Nestin-hRAMP1 and WT littermates (C57BL/6J background)	Russo Lab, Iowa, USA	Male and female, N.R, 20-28 weeks	Perfusion with 4% PFA. Brains were post-fixed for 24 hours in 4% PFA. Cryoprotected in 10%, 20% and 30% sucrose and embedded in OCT. Sectioned at 20 µm.
	Global-hRAMP1 and WT littermates (C57BL/6J background)	Russo Lab, Iowa, USA	Male and female, N.R, 20-28 weeks	Perfusion with 4% PFA. Brains were post-fixed for 24 hours in 4% PFA. Cryoprotected in 10%, 20% and 30% sucrose and embedded in OCT. Sectioned at 10 µm.
	RAMP1 KO and WT littermates (129/S6-SvEv background)	Caron Lab, USA[&]	Male, N.R, 18 weeks (adult mice)	Perfusion with 4% PFA. Brains were post-fixed for 18 hours in 4% PFA. Cryoprotected in 20% sucrose and embedded in OCT. Sectioned at 10 µm.
8 (IHC)	RAMP1 KO and WT littermates (129/S6-SvEv background)	Lutz Lab, Zurich, Switzerland[&]	Male, 22.6-33.0 (g), 18 weeks	Perfused with 4% for 2.5 min. Brains were post-fixed overnight in 4% PFA. Cryoprotected in 20% sucrose and embedded in OCT. Sectioned at 10 µm.
9 (WB)	RAMP1 KO and WT littermates (129/S6-SvEv background)	Lutz Lab, Zurich, Switzerland[&]	Male, 28.0-40.5 (g), 28 weeks	Fresh-frozen in liquid nitrogen. Brains dissected into cerebellum and rest of brain for tissue lysate for western blotting.
	C57BL/6J mice^	Auckland, NZ	Male and female, 24.6-26.8 (g), 13-22 weeks	Fresh-frozen in liquid nitrogen. Brains dissected into cerebellum, brainstem, and cerebrum for tissue lysate for western blotting.

IHC, immunohistochemistry; WB, western blot, SD, Sprague Dawley; NZ, New Zealand; N.R, not recorded; OCT, optimal cutting temperature compound. PFA, paraformaldehyde. *SD rats were obtained from the same colony. ^C57BL/6J mice were obtained from the same colony. [&]Tissues were obtained from two different colonies (Caron and Lutz Lab) but these were the same mouse line that originated from the Caron lab.

Table S5. Secondary antibodies

ICC/IHC	Isotype	Concentration, dilution	Antibody conjugated to dye	Source, Cat#, Lot, RRID
**Anti-goat antibodies**				
Donkey anti-goat 568	IgG (H+L)	2 mg/mL 1:200	AlexaFluor 568	A11057, Lot 1871957, RRID: AB_2534104
Donkey anti-goat 647	IgG (H+L), Cross-adsorbed	2 mg/mL 1:200	AlexaFluor 647 plus	A32849, Lot TF271044, RRID: AB_2762840
Anti-mouse antibody				
Goat anti-mouse 647	IgG (H+L), Cross-adsorbed	2 mg/mL 1:200	AlexaFluor 647	A21235, RRID: AB_2535804
**Anti-rabbit antibodies**				
Goat anti-rabbit 568	IgG (H+L)	2 mg/mL 1:200, 1:1000	AlexaFluor 568	A11011, Lot 1778025, RRID: AB_143157
Donkey anti-rabbit 647	IgG (H+L)	2 mg/mL 1:200, 1:1000	AlexaFluor 647	A31573, Lot 1903516, RRID: AB_2536183
Anti-sheep antibody				
Donkey anti-sheep 647	IgG (H+L)	2 mg/mL 1:200	AlexaFluor 647	A21448, Lot 2045339, RRID: AB_10374882
**Western blot**	**Isotype**	**Concentration, dilution**	**Antibody conjugated to enzyme**	**Source, Cat#, Lot, RRID**
Donkey anti-goat HRP	IgG (H+L), Cross-adsorbed	1 mg/mL 1:2,000, 1:10,000	Horseradish peroxidase	A16005, Lot 6112092917, RRID: AB_2534679
Goat anti-rabbit HRP	IgG, Cross-adsorbed	1 mg/mL 1:2,000, 1:10,000	Horseradish peroxidase	A16110, Lot 47159022717, RRID: AB_2534782
Donkey anti-sheep HRP	IgG Cross-adsorbed	1 mg/mL 1:1,000-1:2,000	Horseradish peroxidase	HAF016, Lot XDP1218091, RRID: AB_562591

Cross-adsorbed antibodies were cross-adsorbed against IgG from a range of species, including rat.

Table S6. Protein amounts loaded for western blotting.

**Antibody characterization in transfected HEK293S cells**	
Human, rat, mouse RAMP1 HEK-293S membrane-enriched	10 µg
**Rat and mouse tissue western blot - 844**	
Rat RAMP1 HEK293S whole cell lysate	1 µg
Rat brain/spleen lysate	100 µg
Mouse RAMP1 HEK-293S whole cell lysate	10 µg
Mouse brain/spleen lysate	100 µg
**Rat and mouse tissue western blot – ab156575**	
Rat RAMP1 HEK293S whole cell lysate	1 µg
Rat brain lysate	100 µg
Rat spleen lysate	50 µg
Mouse RAMP1 HEK-293S whole cell lysate	1 µg
Mouse brain lysate	100 µg
Mouse spleen lysate	20 µg

Supplementary Figures

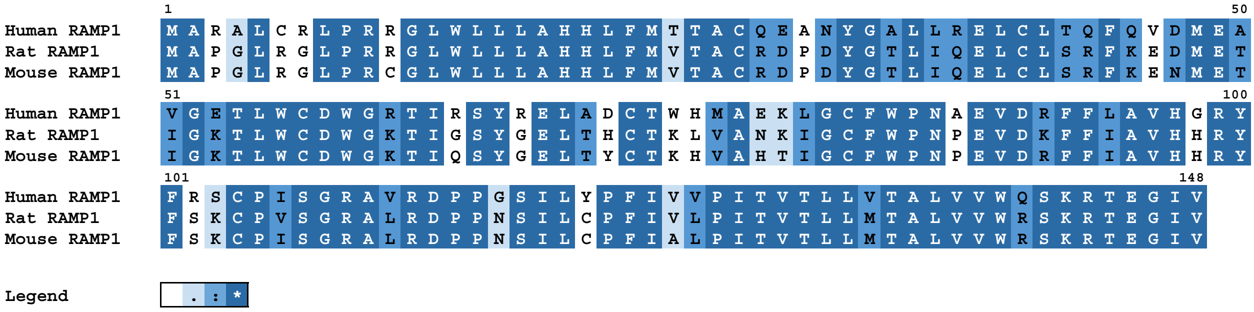

Figure S1. Alignment of RAMP1 sequences from human, rat and mouse. Sequences and amino acid numbering from UniProt were aligned using Clustal Omega 1.2.4 (Sievers et al., 2011; UniProt, 2021). "*" indicates identical residues (dark blue), ":" indicates strong similarity (mid-blue), "." indicates weakly similar residues (light blue) and blank (white) indicates no similarity as determined by Clustal Omega. Uniprot entries were: human RAMP1, O60894; rat RAMP1, Q9JJ74; mouse RAMP1, Q9WTJ5.

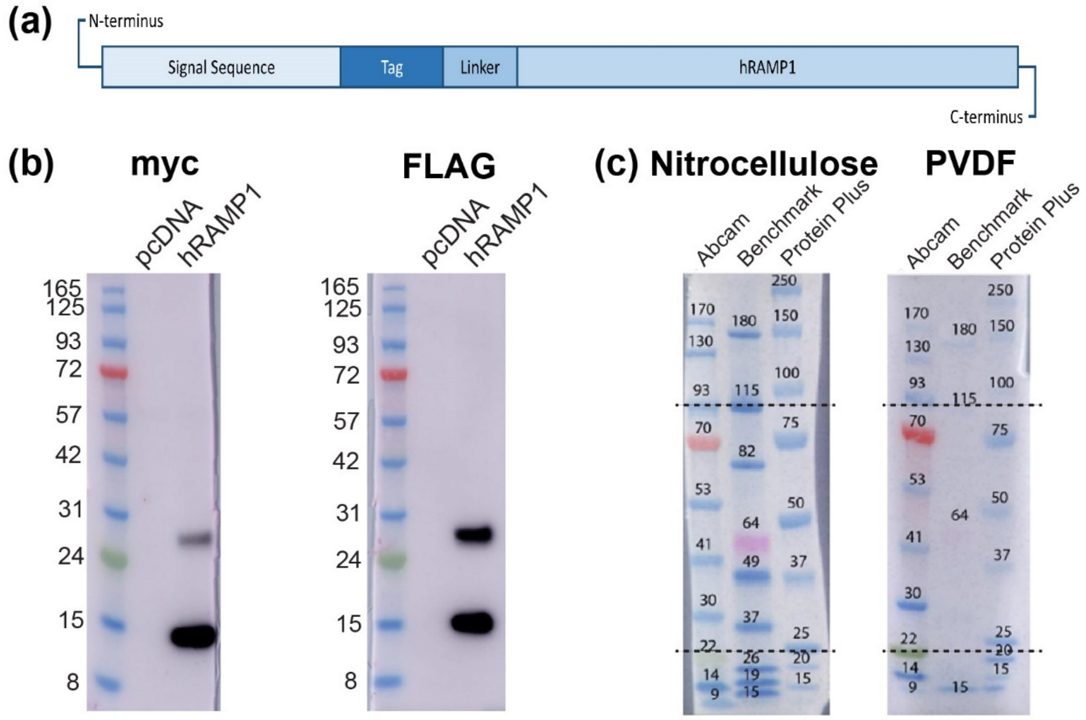

Figure S2. (a) Schematic indicating the location of the myc and FLAG tags within the recombinant RAMP1 sequence. (b) Detection of tagged hRAMP1 by anti-myc (0.8 µg/mL) or anti-FLAG (1 µg/mL) and (c) a direct comparison of protein ladders. (b) Lanes were loaded with 10 µg lysate prepared from HEK293S cells transfected with pcDNA vector or myc- or FLAG-hRAMP1:hCLR. The gels were run with MES buffer and the Abcam protein ladder, and transferred onto a PVDF membrane. Blots were exposed for 2.4s (myc) or 4.5s (FLAG), and are representative of at least three independent experiments. (c) Comparison of the Abcam, Benchmark and Precision Plus protein ladders run using MOPs buffer and transferred onto nitrocellulose or PVDF membrane. Lines highlight discrepancies with the apparent molecular weight between the Benchmark ladder and the Abcam and PrecisionPlus ladders. Republished from (Rees et al., 2022). Differences in apparent molecular weight of Abcam ladder in (b) and (c) due to use of different running buffers (Bass et al., 2017). Black filled arrowheads indicate the predicted monomeric RAMP1 band; red filled arrowheads indicated likely RAMP1 dimers.

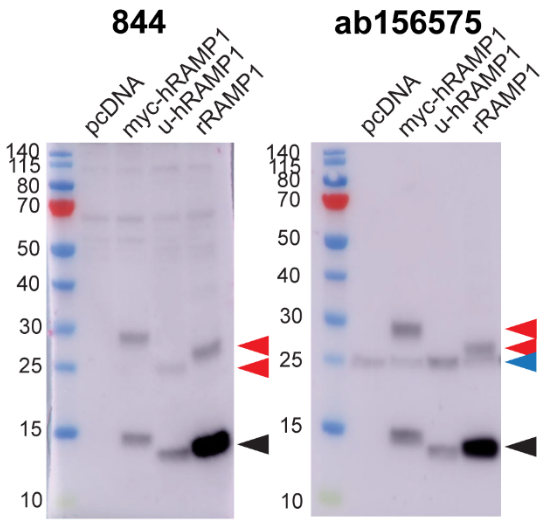

Figure S3. Detection of RAMP1 by 844 (5 µg/mL) and ab156575 (0.612 µg/mL) antibodies run with MES buffer. Lanes contain 10 µg lysate prepared from HEK293S cells transfected with vector (pcDNA) or myc-hRAMP1:hCLR, untagged hRAMP1:hCLR and rRAMP1:rCLR. The blot shows a PageRuler protein ladder (Life Technologies, Cat# 26616) and was exposed for 1.5s (844) or 0.7s (ab156575). The blot is representative of two independent experiments. Black filled arrowheads indicate the predicted monomeric RAMP1 band; red filled arrowheads indicated likely RAMP1 dimers; blue filled arrowheads mark the non-specific ~24 kDa band.

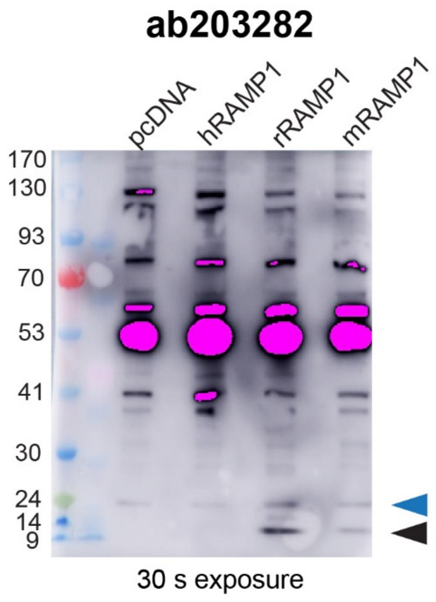

Figure S4. Detection of rat and mouse RAMP1 by ab203282 (1 µg/mL) with a longer exposure. Membrane-enriched protein samples were prepared from HEK293S cells transfected with hCLR:myc-hRAMP1, rCLR:rRAMP1, mCLR:mRAMP1 or vector. The gel was run with an Abcam protein ladder and MOPs buffer and was exposed for 30s. Pink bands indicate signal above the range of the blot imager. Black filled arrowheads indicate the predicted monomeric RAMP1 band; blue filled arrowheads mark the non-specific ~24 kDa band described in the text. The blot is representative of one experiment.

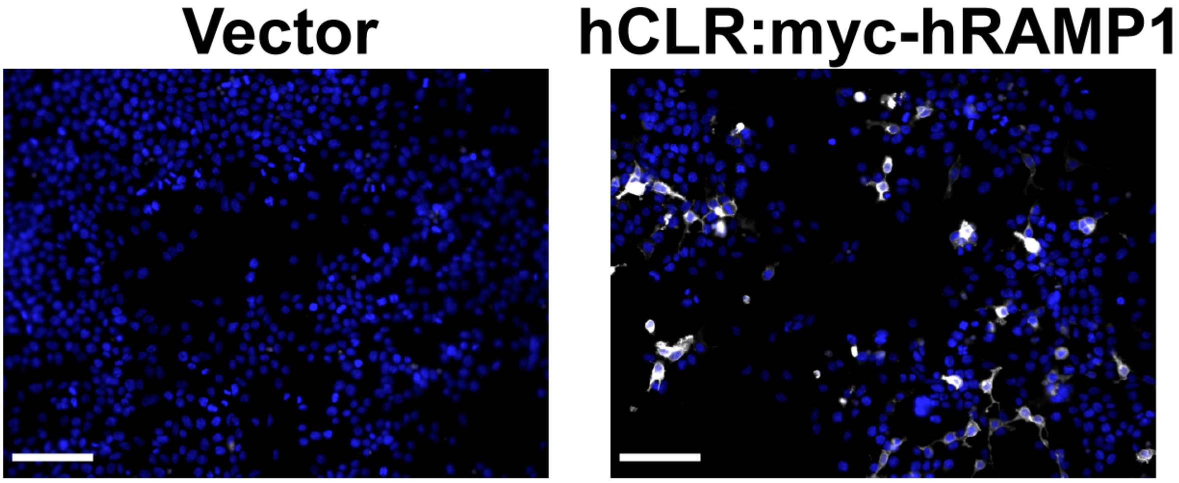

Figure S5. Immunofluorescent ICC of myc-hRAMP1 by the anti-myc antibody (0.8 µg/mL) in transfected HEK293S cells. Immunoreactivity is shown in greyscale and nuclear DAPI shown in blue. Scale bar, 100 µm. Images are representative of at least three independent experiments.

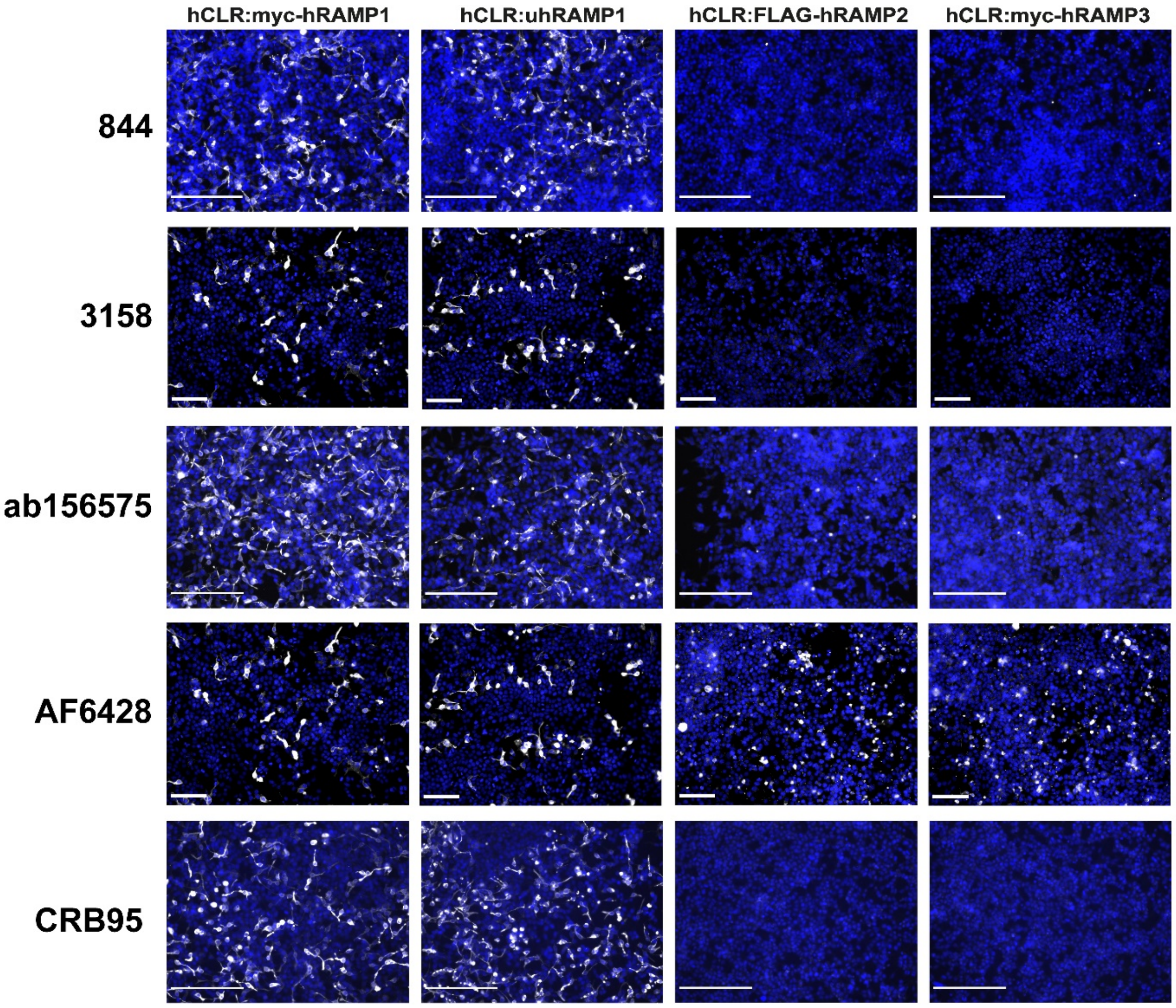

Figure S6. Immunofluorescent ICC of anti-RAMP1 antibodies in transfected HEK293S cells detecting hRAMP1 but not hRAMP2 or hRAMP3. 844 (5 µg/mL), 3158 (1:500), ab156575 (3.06 µg/mL), AF6428 (1 µg/mL), and CRB95 (2 µg/mL) immunoreactivity is shown in greyscale and nuclear DAPI shown in blue. Scale bar, 100 µm (3158/AF6428) or 200 µm (844, ab156575, CRB95). Images are representative of at least three independent experiments. For AF6428, immunofluorescence detected in RAMP2 and RAMP3 transfected cells was comparable to vector control and therefore represents non-specific immunoreactivity.

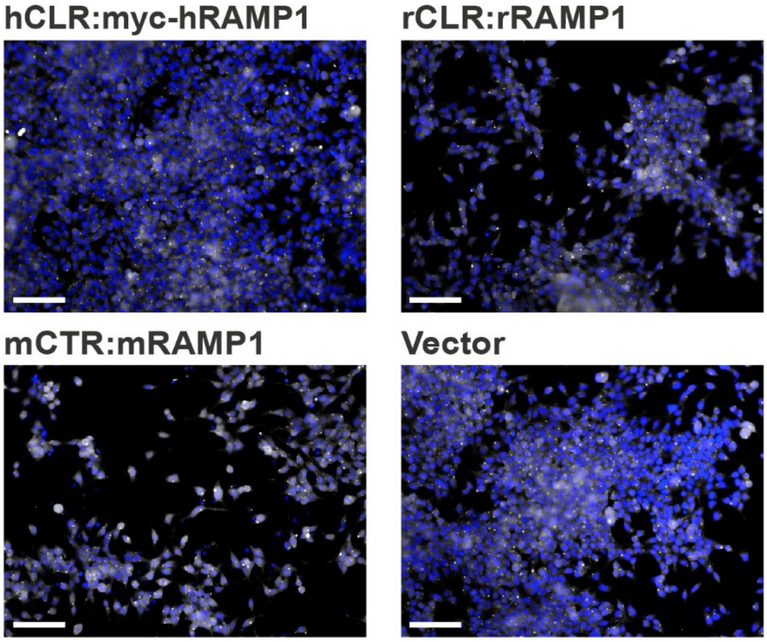

Figure S7. ICC showing lack of immunofluorescence above vector control in transfected HEK293S cells using a higher concentration of anti-RAMP1 antibody PA5-77720 (17 µg/mL). Immunoreactivity is shown in greyscale and nuclear DAPI shown in blue. Images are from one independent experiment. Scale bar, 100 µm.

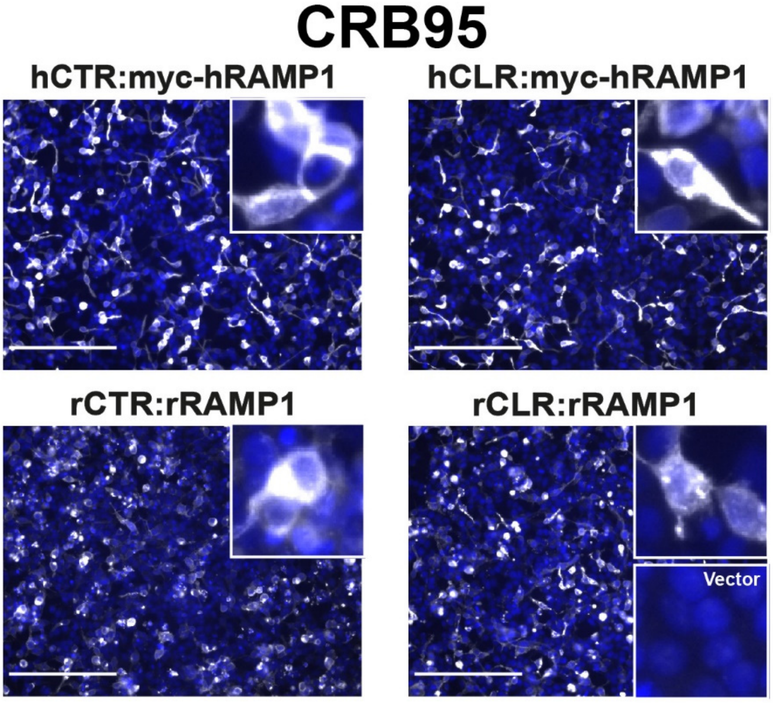

Figure S8. Immunofluorescent ICC of transfected HEK293S cells using the anti-RAMP1 antibody CRB95 (2 µg/mL). Immunoreactivity is shown in greyscale and nuclear DAPI shown in blue. Insets display immunofluorescence for each transfection condition with greater magnification. The lower inset in rCLR:rRAMP1 shows an example of immunoreactivity in the vector control. Images are representative of three independent experiments. Scale bar, 200 µm.

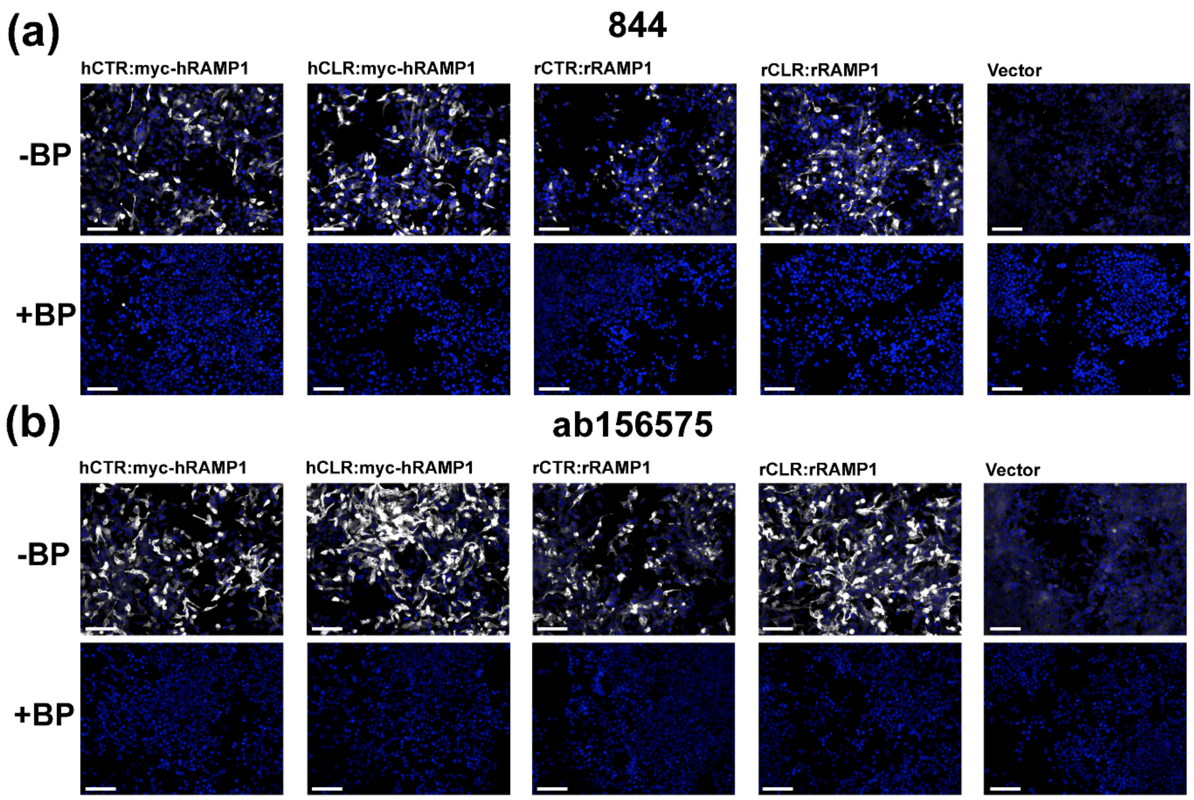

Figure S9. ICC showing blocking peptide (BP) controls for anti-RAMP1 antibodies 844 (5 µg/mL) and ab156575 (3.06 µg/mL) in transiently transfected HEK293S cells. RAMP1-like immunoreactivity for (a) 844 and (b) ab156575 in the absence or presence of 50 µM of blocking peptide. Immunoreactivity is shown in greyscale and nuclear DAPI shown in blue. The intensity was matched for each antibody between the conditions. Scale bar, 100 µm. Although the antigenic sequence for ab156575 was unknown, immunoreactivity appeared to be blocked by same peptide sequence as 844. Images are representative of three independent experiments performed in singlicate or duplicate.

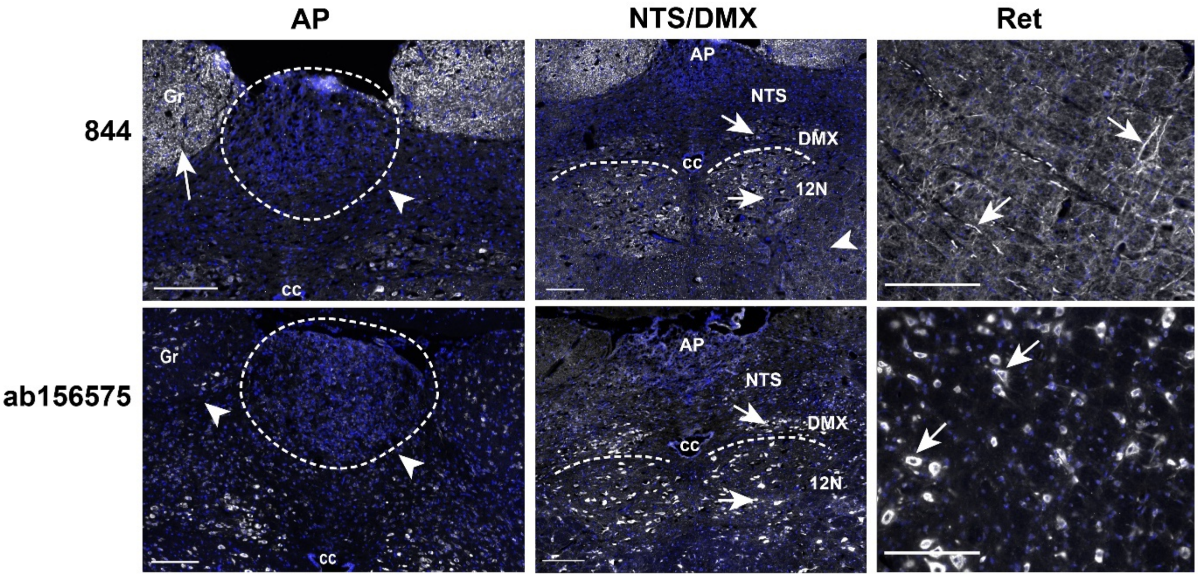

Figure S10. Differential RAMP1-like immunoreactivity in rat brainstem regions by 844 (5 µg/mL) or ab156575 (3.06 µg/mL). Immunoreactivity is shown in greyscale and nuclear DAPI shown in blue. White arrows indicate positively stained cell bodies or fibres and the absence of immunoreactivity is indicated with a white arrowhead. Scale bar, 200 µm. Images are representative of IHC experiments in five (844) or four (ab156575) rats in the AP, NTS/DMX and Ret. Gr = gracile nuclei, AP = area postrema, cc = central canal, NTS = nucleus of the solitary tract, DMX = dorsal motor nucleus of the vagus, 12N = hypoglossal nucleus, Ret = reticular nucleus of the medulla oblongata.

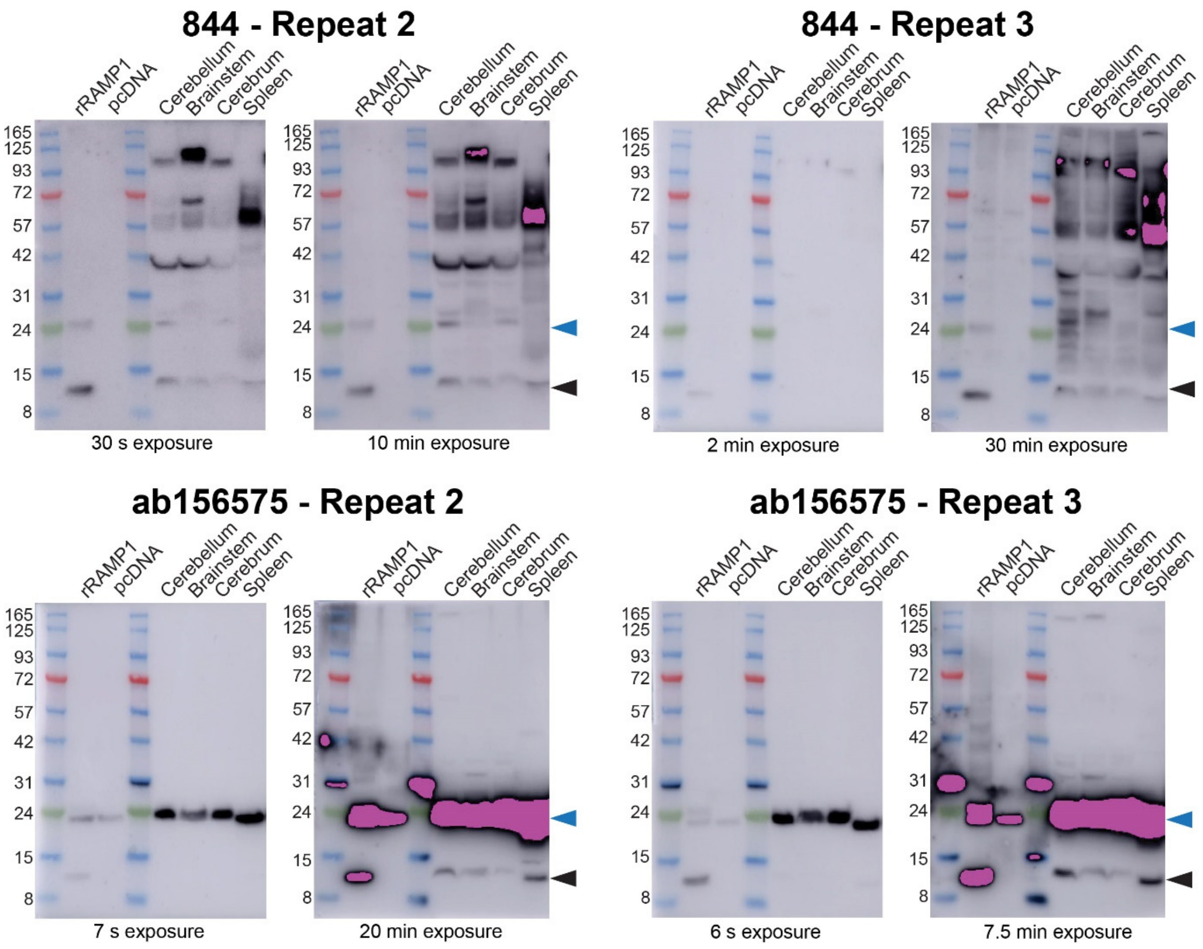

Figure S11. Independent immunoblotting experiments using anti-RAMP1 antibodies 844 (2 µg/mL) and ab156575 (0.612 µg/mL) and rat lysates. Lysates were prepared from HEK293S cells transfected with rCLR:rRAMP1 or vector (pcDNA), or rat brain (cerebellum, brainstem or cerebrum). Exposure times are indicated below each blot. Pink bands indicate signal above the range of the blot imager. Repeat 1 is shown in the main text, Figure 5. Black filled arrowheads indicate the predicted monomeric RAMP1 band; blue filled arrowheads mark the non-specific ~24 kDa band described in the text.

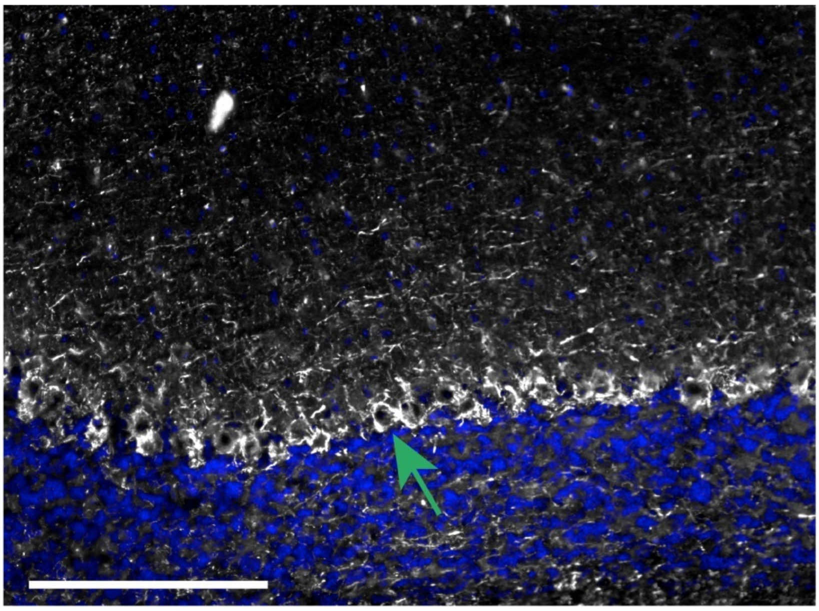

Figure S12. NF200 (2.715 µg/mL) immunofluorescence in C57BL/6J (NZ colony) mouse cerebellum. Immunoreactivity is shown in greyscale and nuclear DAPI shown in blue. The green arrow indicates the granular and molecular layer interface, where most immunofluorescence is present. Scale bar, 200 µm.

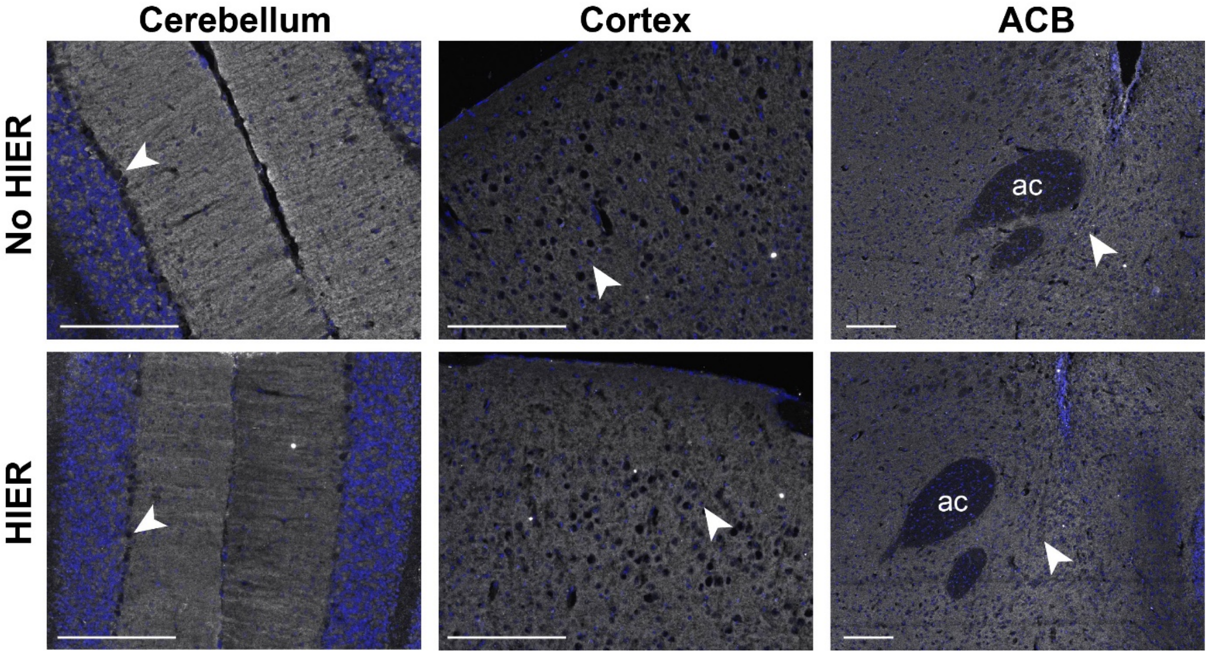

Figure S13. Heat-induced epitope retrieval (HIER) did not reveal 844 (10 µg/µl) immunoreactivity in C57BL/6J (NZ colony) mouse brain regions. Immunoreactivity is shown in greyscale and nuclear DAPI shown in blue. The absence of immunofluorescence is indicated with a white arrowhead. Scale bar, 200 µm. Images are representative of results from three mice. Experiments were performed in parallel for each mouse and the fluorescence intensity is matched for sections treated with HIER and not treated with HIER. ac = anterior commissure. ACB = nucleus accumbens.

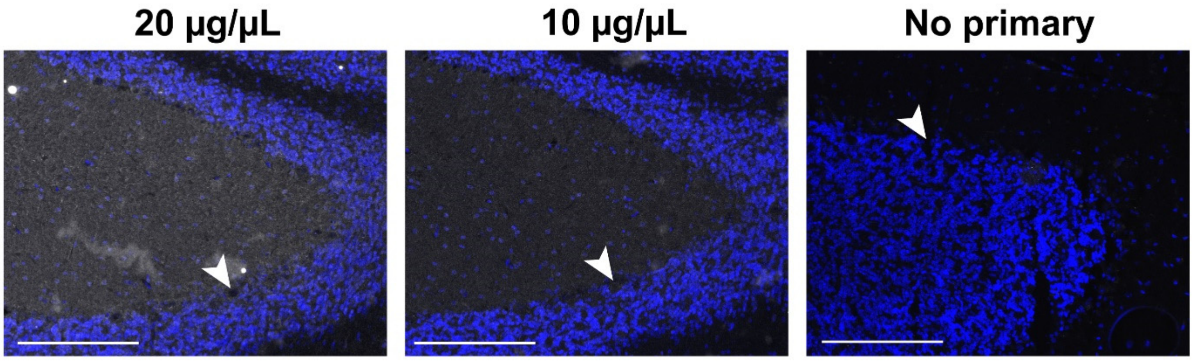

Figure S14. Lack of 844 immunoreactivity at 20 µg/mL and 10 µg/mL in C57BL/6J (NZ colony) mouse cerebellum. Nuclear DAPI shown in blue and lack of immunofluorescence indicated with a white arrowhead. Scale bar, 200 µm. Images are representative of results from three mice. Experiments were performed in parallel for each mouse and the fluorescence intensity is matched for sections incubated with 20 µg/mL and 10 µg/mL of 844 and the no primary control.

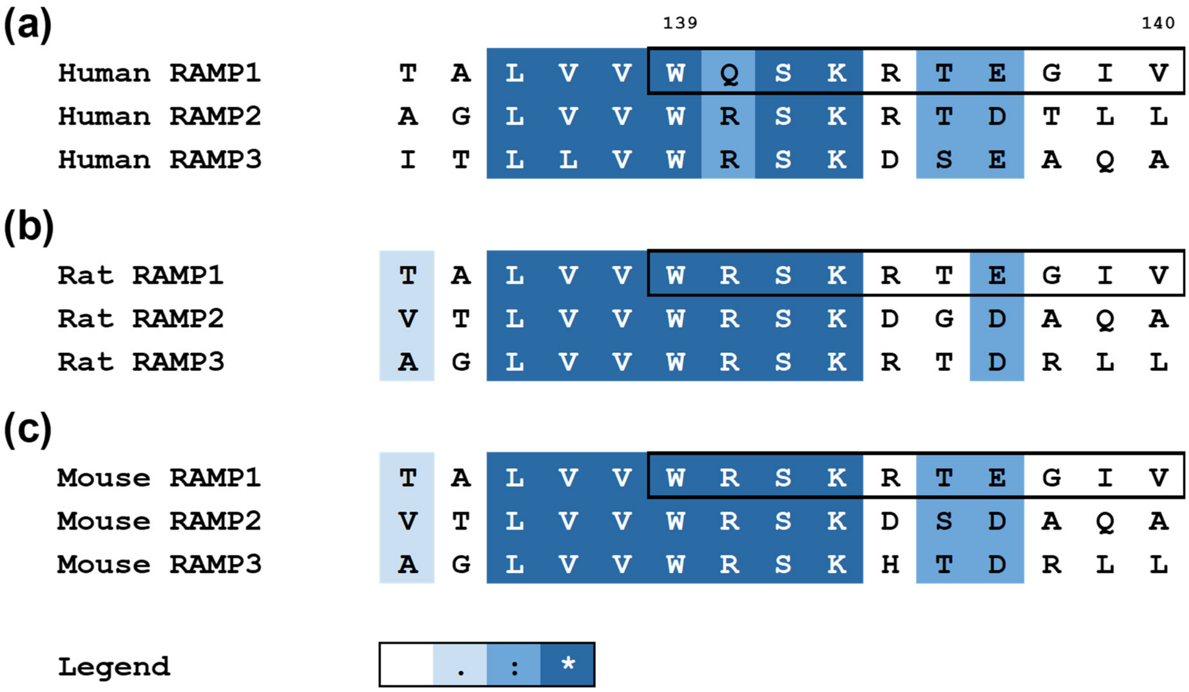

Figure S15. Alignment of RAMP1 C-termini, a region often targeted by anti-RAMP1 antibodies, with RAMP2 and RAMP3 C-termini. Sequences and amino acid numbering from UniProt were aligned using Clustal Omega (Sievers et al., 2011; UniProt, 2021) for a) human, b) rat and c) mouse RAMPs. Colours correspond to residue similarity, "*" indicates identical residues (dark blue), ":" indicates strong similarity (mid-blue), "." indicates weakly similar (light blue) and blank (white) indicates no similarity as determined by Clustal Omega. The black box highlights the C-terminus (residues 139-148) of RAMP1 for human, rat and mouse.

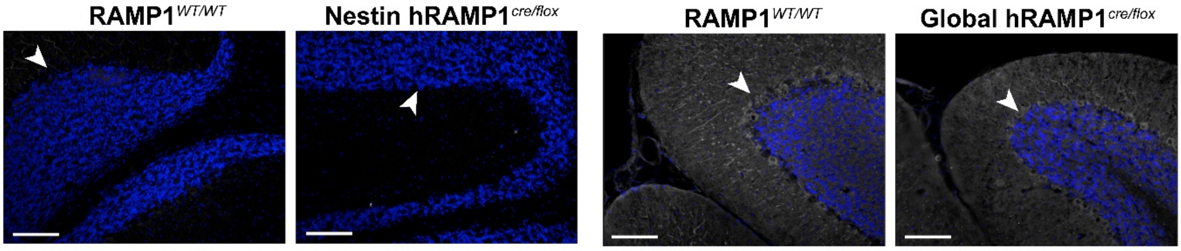

Figure S16. Lack of detectable RAMP1-like immunoreactivity by AF6428 (1 µg/mL) in mouse hRAMP1 overexpression cerebellum. Immunoreactivity is shown in greyscale and nuclear DAPI shown in blue. The absence of immunofluorescence is indicated with a white arrowhead. Scale bar, 100 µm. Images are representative of results from one (Nestin-hRAMP1) or three (Global-hRAMP1) mice.

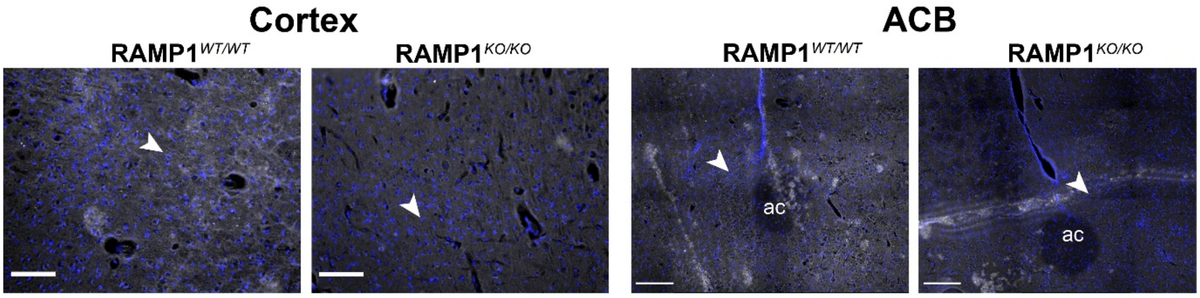

Figure S17. Lack of detectable RAMP1-like immunoreactivity by ab203282 (5 µg/mL) in mouse WT and KO cortex and cerebellum. Immunoreactivity is shown in greyscale and nuclear DAPI shown in blue. The absence of immunoreactivity is indicated with a white arrowhead. Scale bar, 100 µm (cortex) and 200 µm (ACB). Images are representative of results from two mice.

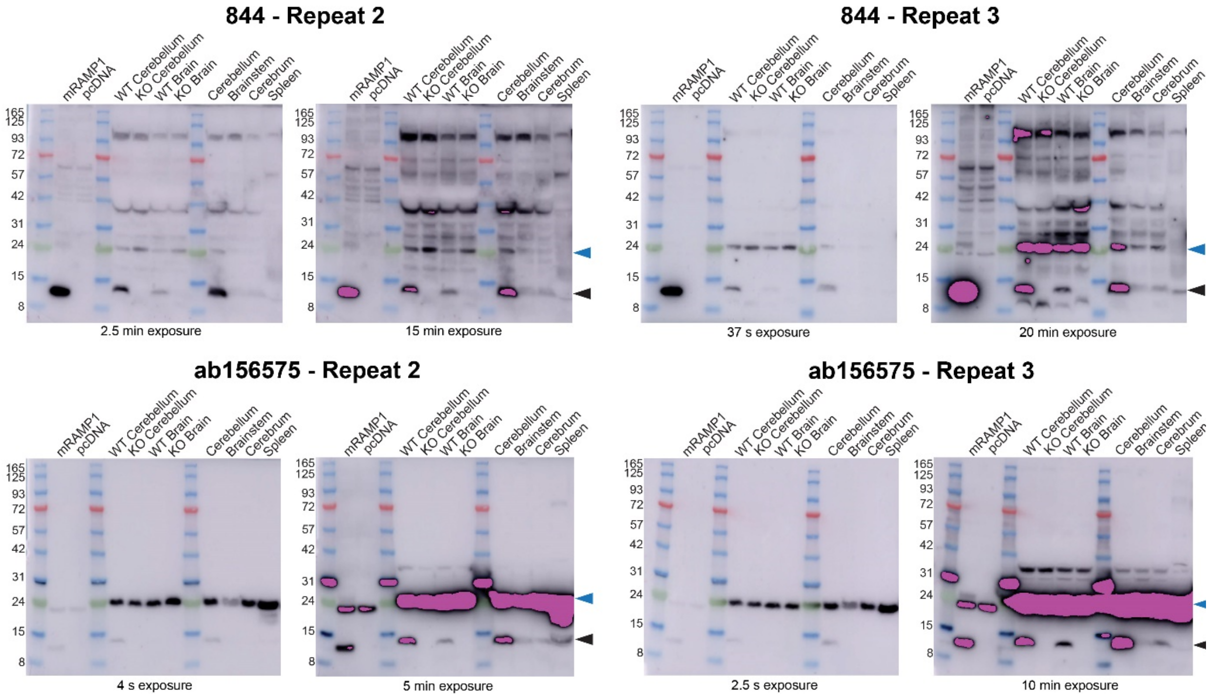

Figure S18. Independent immunoblotting experiments using anti-RAMP1 antibodies 844 (2 µg/mL) and ab156575 (0.612 µg/mL) and mouse lysates. Lysates were prepared from HEK293S cells transfected with mCLR:mRAMP1 or vector (pcDNA), or littermate mouse WT and KO cerebellum and brain excluding cerebellum, or NZ mouse cerebellum, brainstem, and cerebrum. Exposure times are indicated below each blot. Pink bands indicate signal above the range of the blot imager. Repeat 1 is shown in the main text, Figure 9. Black filled arrowheads indicate the predicted monomeric RAMP1 band; blue filled arrowheads mark the non-specific ~24 kDa band described in the text.

Supplemental Chemistry

General information for anti-RAMP1 blocking peptide

The sequence for the blocking peptide is shown in Figure 1 (844 antigen) and is also present in Table S2.

All reagents were purchased as reagent grade and used without further purification. Semi-preparative RP- HPLC was performed on a Thermo Scientific (Waltham, MA) Dionex Ultimate 3000 HPLC equipped with a four channel UV Detector at 210, 225, 254 and 280 nm using either an analytical column (Waters (Milford, MA) XTerra® MS C18, (5 µm; 4.6 × 150 mm) at a flow rate of 1 mL min-1 or a semi-preparative column (Phenomenex® Gemini C18, (5 µm; 10 × 250 mm) at a flow rate of 5 mL min-1. A suitably adjusted gradient of 5% B to 95% B was used, where solvent A was 0.1% TFA in H_2O and B was 0.1% TFA in acetonitrile. Solid phase peptide synthesis was performed using a Biotage® Initiator Alstra. LCMS spectra were acquired on either an Agilent Technologies (Santa Clara, CA)1120 Compact LC equipped with a Hewlett-Packard (Palo Alto, CA) 1100 MSD mass spectrometer or an Agilent Technologies 1260 Infinity LC equipped with an Agilent Technologies 6120 Quadrupole mass spectrometer. An analytical column (Agilent C3, 3.5 µm; 3.0 × 150 mm) was used at a flow rate of 0.3 mL min-1 using a linear gradient of 5% B to 95% B over 30 min, where solvent A was 0.1% formic acid in H_2O and B was 0.1% formic acid in acetonitrile. High resolution mass spectra (HRMS) were obtained using the micrOTOF-Q spectrometer operating at a nominal accelerating voltage of 70 eV. Analyses are shown in Figures S19-S21.

HPLC of blank sample in TFA buffer solution at 3% B/min

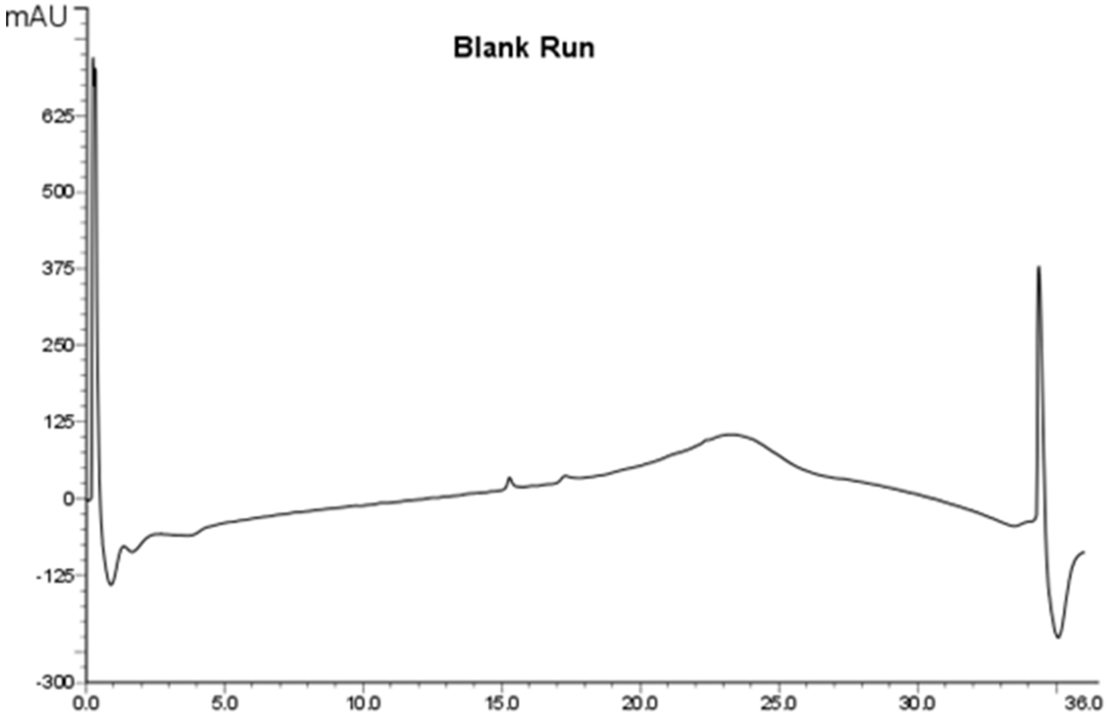

Figure S19. Analytical RP-HPLC chromatogram for blank sample. Chromatographic separations were performed on a Thermo Scientific (Waltham, MA) Dionex Ultimate 3000 HPLC using a XTerra® MS C18 column (5 µm; 4.6 × 150 mm) and a linear gradient of 5-95% B in 35 min at room temperature, *ca*. 3% B per min at a flow rate of 1.0 mL/min. Buffer A: H_2O containing 0.1% TFA (*v*/*v*); Buffer B: acetonitrile containing 0.1% TFA (*v*/*v*).

HPLC of peptide 844 in TFA buffer solution at 3% B/min

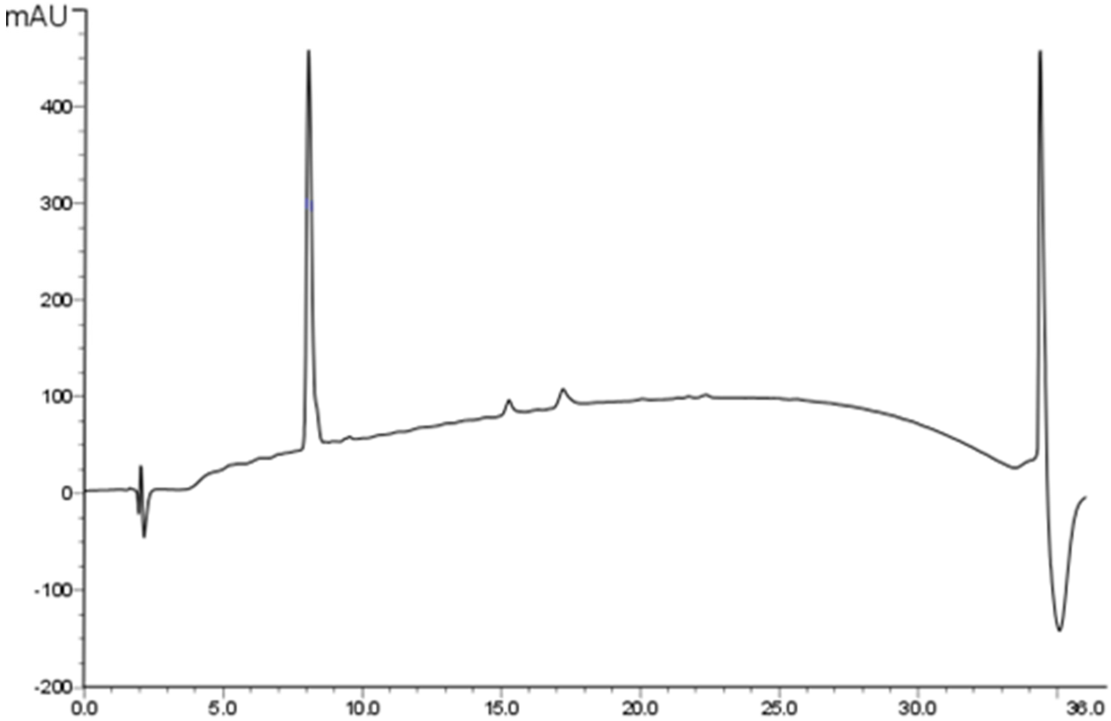

Figure S20. Analytical RP-HPLC chromatogram for blocking peptide; t_R = 8.1 min (> 98% purity as judged by peak area of RP-HPLC at 210 nm). Chromatographic separations were performed on a Thermo Scientific (Waltham, MA) Dionex Ultimate 3000 HPLC using a XTerra® MS C18 column (5 µm; 4.6 × 150 mm) and a linear gradient of 5-95% B in 35 min at room temperature, *ca*. 3% B per min at a flow rate of 1.0 mL/min. Buffer A: H_2O containing 0.1% TFA (*v*/*v*); Buffer B: acetonitrile containing 0.1% TFA (*v*/*v*).

ESI-MS of peptide 844

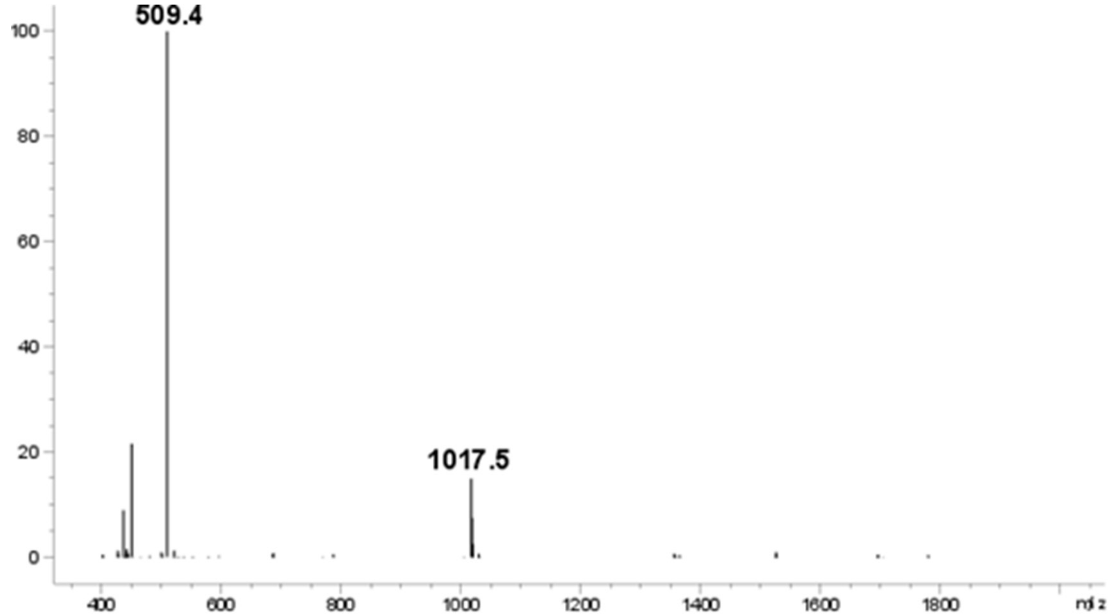

Figure S21. ESI-MS of purified Peptide 844 17/7/19. (*m/z* $[M+1H]^{1+}$ calcd: 1018.1; found: 1017.5; $[M+2H]^{2+}$ calcd: 509.5; found: 509.4. Mass deconvolution calculated at 1016.65 Da with standard deviation of 0.21; theoretical mass calculated at 1017.15 Da.

References:

Aranas, C., Vestlund, J., Witley, S., Edvardsson, C. E., Kalafateli, A. L., & Jerlhag, E. (2021). Salmon Calcitonin Attenuates Some Behavioural Responses to Nicotine in Male Mice. *Front Pharmacol, 12*, 685631. 10.3389/fphar.2021.685631

Bass, J. J., Wilkinson, D. J., Rankin, D., Phillips, B. E., Szewczyk, N. J., Smith, K., & Atherton, P. J. (2017). An overview of technical considerations for Western blotting applications to physiological research. *Scand J Med Sci Sports, 27*(1), 4-25. 10.1111/sms.12702

Blixt, F. W., Radziwon-Balicka, A., Edvinsson, L., & Warfvinge, K. (2017). Distribution of CGRP and its receptor components CLR and RAMP1 in the rat retina. *Exp Eye Res, 161*, 124-131. 10.1016/j.exer.2017.06.002

Bohn, K. J., Li, B., Huang, X., Mason, B. N., Wattiez, A. S., Kuburas, A., Walker, C. S., Yang, P., Yu, J., Heinz, B. A., Johnson, K. W., & Russo, A. F. (2017). CGRP receptor activity in mice with global expression of human receptor activity modifying protein 1. *Br J Pharmacol, 174*(12), 1826-1840. 10.1111/bph.13783

Coester, B., Pence, S. W., Arrigoni, S., Boyle, C. N., Le Foll, C., & Lutz, T. A. (2020). RAMP1 and RAMP3 Differentially Control Amylin's Effects on Food Intake, Glucose and Energy Balance in Male and Female Mice. *Neuroscience, 447*, 74-93. 10.1016/j.neuroscience.2019.11.036

Csati, A., Tajti, J., Tuka, B., Edvinsson, L., & Warfvinge, K. (2012). Calcitonin gene-related peptide and its receptor components in the human sphenopalatine ganglion -- interaction with the sensory system. *Brain Res, 1435*, 29-39. 10.1016/j.brainres.2011.11.058

De Logu, F., Nassini, R., Hegron, A., Landini, L., Jensen, D. D., Latorre, R., Ding, J., Marini, M., Souza Monteiro de Araujo, D., Ramirez-Garcia, P., Whittaker, M., Retamal, J., Titiz, M., Innocenti, A., Davis, T. P., Veldhuis, N., Schmidt, B. L., Bunnett, N. W., & Geppetti, P. (2022). Schwann cell endosome CGRP signals elicit periorbital mechanical allodynia in mice. *Nat Commun, 13*(1), 646. 10.1038/s41467-022-28204-z

Edvinsson, J. C. A., Warfvinge, K., Krause, D. N., Blixt, F. W., Sheykhzade, M., Edvinsson, L., & Haanes, K. A. (2019). C-fibers may modulate adjacent Adelta-fibers through axon-axon CGRP signaling at nodes of Ranvier in the trigeminal system. *J Headache Pain, 20*(1), 105. 10.1186/s10194-019-1055-3

Edvinsson, L., Eftekhari, S., Salvatore, C. A., & Warfvinge, K. (2011). Cerebellar distribution of calcitonin gene-related peptide (CGRP) and its receptor components calcitonin receptor-like receptor (CLR) and receptor activity modifying protein 1 (RAMP1) in rat. *Mol Cell Neurosci, 46*(1), 333-339. 10.1016/j.mcn.2010.10.005

Edvinsson, L., Grell, A. S., & Warfvinge, K. (2020). Expression of the CGRP Family of Neuropeptides and their Receptors in the Trigeminal Ganglion. *J Mol Neurosci, 70*(5), 930–944. 10.1007/s12031-020-01493-z

Eftekhari, S., & Edvinsson, L. (2011). Calcitonin gene-related peptide (CGRP) and its receptor components in human and rat spinal trigeminal nucleus and spinal cord at C1-level. *BMC Neurosci, 12*, 112. 10.1186/1471-2202-12-112

Eftekhari, S., Salvatore, C. A., Calamari, A., Kane, S. A., Tajti, J., & Edvinsson, L. (2010). Differential distribution of calcitonin gene-related peptide and its receptor components in the human trigeminal ganglion. *Neuroscience, 169*(2), 683-696. 10.1016/j.neuroscience.2010.05.016

Eftekhari, S., Salvatore, C. A., Gaspar, R. C., Roberts, R., O'Malley, S., Zeng, Z., & Edvinsson, L. (2013). Localization of CGRP receptor components, CGRP, and receptor binding sites in human and rhesus cerebellar cortex. *Cerebellum, 12*(6), 937-949. 10.1007/s12311-013-0509-4

Eftekhari, S., Salvatore, C. A., Johansson, S., Chen, T. B., Zeng, Z., & Edvinsson, L. (2015). Localization of CGRP, CGRP receptor, PACAP and glutamate in trigeminal ganglion. Relation to the blood-brain barrier. *Brain Res, 1600*, 93-109. 10.1016/j.brainres.2014.11.031

Eftekhari, S., Warfvinge, K., Blixt, F. W., & Edvinsson, L. (2013). Differentiation of nerve fibers storing CGRP and CGRP receptors in the peripheral trigeminovascular system. *J Pain, 14*(11), 1289-1303. 10.1016/j.jpain.2013.03.010

Ernstsen, C., Christensen, S. L., Rasmussen, R. H., Nielsen, B. S., Jansen-Olesen, I., Olesen, J., & Kristensen, D. M. (2022). The PACAP pathway is independent of CGRP in mouse models of migraine: possible new drug target? *Brain* 10.1093/brain/awac040

Filiz, A., Tepe, N., Eftekhari, S., Boran, H. E., Dilekoz, E., Edvinsson, L., & Bolay, H. (2019). CGRP receptor antagonist MK-8825 attenuates cortical spreading depression induced pain behavior. *Cephalalgia, 39*(3), 354-365. 10.1177/0333102417735845

Ghanizada, H., Al-Karagholi, M. A., Walker, C. S., Arngrim, N., Rees, T., Petersen, J., Siow, A., Morch-Rasmussen, M., Tan, S., O'Carroll, S. J., Harris, P., Skovgaard, L. T., Jorgensen, N. R., Brimble, M., Waite, J. S., Rea, B. J., Sowers, L. P., Russo, A. F., Hay, D. L., & Ashina, M. (2021). Amylin analog pramlintide induces migraine-like attacks in patients. *Ann Neurol, 89*(6), 1157-1171. 10.1002/ana.26072

Johnson, J. (2012). Not seeing is not believing: improving the visibility of your fluorescence images. *Mol Biol Cell, 23*(5), 754-757. 10.1091/mbc.E11-09-0824

Kang, Y., Ding, L., Dai, H., Wang, F., Zhou, H., Gao, Q., Xiong, X., Zhang, F., Song, T., Yuan, Y., Zhu, G., & Zhou, Y. (2019). Intermedin in Paraventricular Nucleus Attenuates Ang II-Induced Sympathoexcitation through the Inhibition of NADPH Oxidase-Dependent ROS Generation in Obese Rats with Hypertension. *Int J Mol Sci, 20*(17) 10.3390/ijms20174217

Lein, E. S., Hawrylycz, M. J., Ao, N., Ayres, M., Bensinger, A., Bernard, A., Boe, A. F., Boguski, M. S., Brockway, K. S., Byrnes, E. J., Chen, L., Chen, L., Chen, T. M., Chin, M. C., Chong, J., Crook, B. E., Czaplinska, A., Dang, C. N., Datta, S., Dee, N. R., Desaki, A. L., Desta, T., Diep, E., Dolbeare, T. A., Donelan, M. J., Dong, H. W., Dougherty, J. G., Duncan, B. J., Ebbert, A. J., Eichele, G., Estin, L. K., Faber, C., Facer, B. A., Fields, R., Fischer, S. R., Fliss, T. P., Frensley, C., Gates, S. N., Glattfelder, K. J., Halverson, K. R., Hart, M. R., Hohmann, J. G., Howell, M. P., Jeung, D. P., Johnson, R. A., Karr, P. T., Kawal, R., Kidney, J. M., Knapik, R. H., Kuan, C. L., Lake, J. H., Laramee, A. R., Larsen, K. D., Lau, C., Lemon, T. A., Liang, A. J., Liu, Y., Luong, L. T., Michaels, J., Morgan, J. J., Morgan, R. J., Mortrud, M. T., Mosqueda, N. F., Ng, L. L., Ng, R., Orta, G. J., Overly, C. C., Pak, T. H., Parry, S. E., Pathak, S. D., Pearson, O. C., Puchalski, R. B., Riley, Z. L., Rockett, H. R., Rowland, S. A., Royall, J. J., Ruiz, M. J., Sarno, N. R., Schaffnit, K., Shapovalova, N. V., Sivisay, T., Slaughterbeck, C. R., Smith, S. C., Smith, K. A., Smith, B. I., Sodt, A. J., Stewart, N. N., Stumpf, K. R., Sunkin, S. M., Sutram, M., Tam, A., Teemer, C. D., Thaller, C., Thompson, C. L., Varnam, L. R., Visel, A., Whitlock, R. M., Wohnoutka, P. E., Wolkey, C. K., Wong, V. Y., Wood, M., Yaylaoglu, M. B., Young, R. C., Youngstrom, B. L., Yuan, X. F., Zhang, B., Zwingman, T. A., & Jones, A. R. (2007). Genome-wide atlas of gene expression in the adult mouse brain. *Nature, 445*(7124), 168-176. 10.1038/nature05453

Li, M., Wetzel-Strong, S. E., Hua, X., Tilley, S. L., Oswald, E., Krummel, M. F., & Caron, K. M. (2014). Deficiency of RAMP1 attenuates antigen-induced airway hyperresponsiveness in mice. *PLoS One, 9*(7), e102356. 10.1371/journal.pone.0102356

Moye, L. S., Siegersma, K., Dripps, I., Witkowski, W., Mangutov, E., Wang, D., Scherrer, G., & Pradhan, A. A. (2021). Delta opioid receptor regulation of calcitonin gene-related peptide dynamics in the trigeminal complex. *Pain, 162*(8), 2297-2308. 10.1097/j.pain.0000000000002235

Pozo-Rosich, P., Storer, R. J., Charbit, A. R., & Goadsby, P. J. (2015). Periaqueductal gray calcitonin gene-related peptide modulates trigeminovascular neurons. *Cephalalgia, 35*(14), 1298-1307. 10.1177/0333102415576723

Preibisch, S., Saalfeld, S., & Tomancak, P. (2009). Globally optimal stitching of tiled 3D microscopic image acquisitions. *Bioinformatics, 25*(11), 1463-1465. 10.1093/bioinformatics/btp184

Rees, T. A., Russo, A. F., O'Carroll, S. J., Hay, D. L., & Walker, C. S. (2022). CGRP and the Calcitonin Receptor are Co-Expressed in Mouse, Rat and Human Trigeminal Ganglia Neurons. *Frontiers in Physiology, 13* 10.3389/fphys.2022.860037

Seiler, K., Nusser, J. I., Lennerz, J. K., Neuhuber, W. L., & Messlinger, K. (2013). Changes in calcitonin gene-related peptide (CGRP) receptor component and nitric oxide receptor (sGC) immunoreactivity in rat trigeminal ganglion following glyceroltrinitrate pretreatment. *J Headache Pain, 14*, 74. 10.1186/1129-2377-14-74

Sheykhzade, M., Amandi, N., Pla, M. V., Abdolalizadeh, B., Sams, A., Warfvinge, K., Edvinsson, L., & Pickering, D. S. (2017). Binding and functional pharmacological characteristics of gepant-type antagonists in rat brain and mesenteric arteries. *Vascul Pharmacol, 90*, 36-43. 10.1016/j.vph.2017.02.001

Sievers, F., Wilm, A., Dineen, D., Gibson, T. J., Karplus, K., Li, W., Lopez, R., McWilliam, H., Remmert, M., Soding, J., Thompson, J. D., & Higgins, D. G. (2011). Fast, scalable generation of high-quality protein multiple sequence alignments using Clustal Omega. *Mol Syst Biol, 7*, 539. 10.1038/msb.2011.75

Summ, O., Charbit, A. R., Andreou, A. P., & Goadsby, P. J. (2010). Modulation of nocioceptive transmission with calcitonin gene-related peptide receptor antagonists in the thalamus. *Brain, 133*(9), 2540-2548. 10.1093/brain/awq224

Uhlen, M., Fagerberg, L., Hallstrom, B. M., Lindskog, C., Oksvold, P., Mardinoglu, A., Sivertsson, A., Kampf, C., Sjostedt, E., Asplund, A., Olsson, I., Edlund, K., Lundberg, E., Navani, S., Szigyarto, C. A., Odeberg, J., Djureinovic, D., Takanen, J. O., Hober, S., Alm, T., Edqvist, P. H., Berling, H., Tegel, H., Mulder, J., Rockberg, J., Nilsson, P., Schwenk, J. M., Hamsten, M., von Feilitzen, K., Forsberg, M., Persson, L., Johansson, F., Zwahlen, M., von Heijne, G., Nielsen, J., & Ponten, F. (2015). Proteomics. Tissue-based map of the human proteome. *Science, 347*(6220), 1260419. 10.1126/science.1260419

UniProt, C. (2021). UniProt: the universal protein knowledgebase in 2021. *Nucleic Acids Res, 49*(D1), D480-D489. 10.1093/nar/gkaa1100

Vilotti, S., Vana, N., Van den Maagdenberg, A. M., & Nistri, A. (2016). Expression and function of calcitonin gene-related peptide (CGRP) receptors in trigeminal ganglia of R192Q Cacna1a knock-in mice. *Neurosci Lett, 620*, 104-110. 10.1016/j.neulet.2016.03.046

Walker, C. S., Eftekhari, S., Bower, R. L., Wilderman, A., Insel, P. A., Edvinsson, L., Waldvogel, H. J., Jamaluddin, M. A., Russo, A. F., & Hay, D. L. (2015). A second trigeminal CGRP receptor: function and expression of the AMY1 receptor. *Ann Clin Transl Neurol, 2*(6), 595-608. 10.1002/acn3.197

Warfvinge, K., & Edvinsson, L. (2019). Distribution of CGRP and CGRP receptor components in the rat brain. *Cephalalgia, 39*(3), 342-353. 10.1177/0333102417728873

Warfvinge, K., Krause, D., & Edvinsson, L. (2020). The distribution of oxytocin and the oxytocin receptor in rat brain: relation to regions active in migraine. *J Headache Pain, 21*(1), 10. 10.1186/s10194-020-1079-8

Xu, X., Cai, X., Liu, X., & Guo, S. W. (2021). Possible involvement of neuropeptide and neurotransmitter receptors in Adenomyosis. *Reprod Biol Endocrinol, 19*(1), 25. 10.1186/s12958-021-00711-6

Zhang, Z., Winborn, C. S., Marquez de Prado, B., & Russo, A. F. (2007). Sensitization of calcitonin gene-related peptide receptors by receptor activity-modifying protein-1 in the trigeminal ganglion. *J Neurosci, 27*(10), 2693-2703. 10.1523/JNEUROSCI.4542-06.2007

Zhou, X. Y., Xu, X. M., Wu, S. Y., Wang, F., Zhang, Z. C., Yang, Y. L., Li, M., & Wei, X. Z. (2017). Low-Intensity Pulsed Ultrasound-Induced Spinal Fusion is Coupled with Enhanced Calcitonin Gene-Related Peptide Expression in Rat Model. *Ultrasound Med Biol, 43*(7), 1486-1493. 10.1016/j.ultrasmedbio.2017.03.012
